# Structure-Activity Relationship Analysis of 3-Phenylcoumarin-Based Monoamine Oxidase B Inhibitors

**DOI:** 10.3389/fchem.2018.00041

**Published:** 2018-03-02

**Authors:** Sanna Rauhamäki, Pekka A. Postila, Sanna Niinivehmas, Sami Kortet, Emmi Schildt, Mira Pasanen, Elangovan Manivannan, Mira Ahinko, Pasi Koskimies, Niina Nyberg, Pasi Huuskonen, Elina Multamäki, Markku Pasanen, Risto O. Juvonen, Hannu Raunio, Juhani Huuskonen, Olli T. Pentikäinen

**Affiliations:** ^1^Computational Bioscience Laboratory, Department of Biological and Environmental Science & Nanoscience Center, University of Jyväskylä, Jyväskylä, Finland; ^2^Department of Chemistry & Nanoscience Center, University of Jyväskylä, Jyväskylä, Finland; ^3^School of Pharmacy, Devi Ahilya University, Madhya Pradesh, India; ^4^Forendo Pharma Ltd., Turku, Finland; ^5^School of Pharmacy, University of Eastern Finland, Kuopio, Finland; ^6^MedChem.fi, Institute of Biomedicine, University of Turku, Turku, Finland

**Keywords:** 3-phenylcoumarin, monoamine oxidase B (MAO-B), structure-activity relationship (SAR), virtual drug design, Parkinson's disease

## Abstract

Monoamine oxidase B (MAO-B) catalyzes deamination of monoamines such as neurotransmitters dopamine and norepinephrine. Accordingly, small-molecule MAO-B inhibitors potentially alleviate the symptoms of dopamine-linked neuropathologies such as depression or Parkinson's disease. Coumarin with a functionalized 3-phenyl ring system is a promising scaffold for building potent MAO-B inhibitors. Here, a vast set of 3-phenylcoumarin derivatives was designed using virtual combinatorial chemistry or rationally *de novo* and synthesized using microwave chemistry. The derivatives inhibited the MAO-B at 100 nM−1 μM. The IC_50_ value of the most potent derivative **1** was 56 nM. A docking-based structure-activity relationship analysis summarizes the atom-level determinants of the MAO-B inhibition by the derivatives. Finally, the cross-reactivity of the derivatives was tested against monoamine oxidase A and a specific subset of enzymes linked to estradiol metabolism, known to have coumarin-based inhibitors. Overall, the results indicate that the 3-phenylcoumarins, especially derivative **1**, present unique pharmacological features worth considering in future drug development.

## Introduction

During neuronal signaling, neurotransmitters are released from the presynaptic cell into the synaptic cleft, from where they bind into their specific receptors embedded on the postsynaptic membrane. The membrane lipid bilayer, especially its anionic phospholipid constituents, has been suggested to play a role in the small-molecule entry processes with the receptors (Orłowski et al., 2012; Postila et al., 2016; Mokkila et al., 2017). Moreover, to assure that the neurotransmission remains transient, the neurotransmitters are removed quickly from the synaptic cleft via enzymatic degradation and cellular uptake.

**Figure 1:**
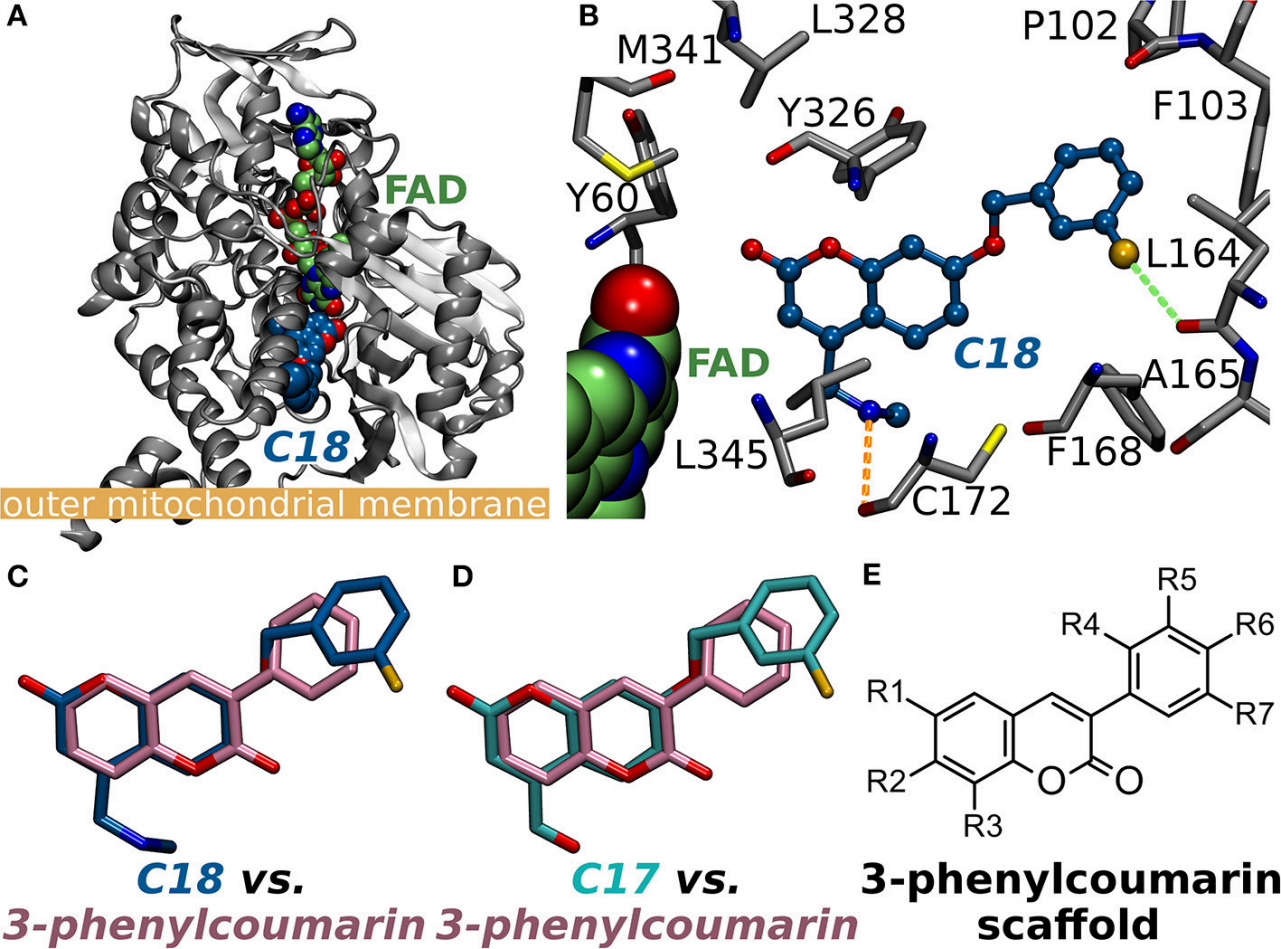
Monoamine oxidase B structure and the small-molecule inhibitors. **(A)** The cytoplasmic MAO-B monomer (gray cartoon; PDB: 2V61; A-chain) (Binda et al., 2007) is anchored by its C-terminal helix onto the outer mitochondrial membrane [thick orange line; from the OPM database (Lomize et al., 2006)]. The bound inhibitor 7-(3-chlorobenzyloxy)-4-(methylamino)methyl-coumarin (*C18* in PDB: 2V61; blue backbone) and the cofactor flavin adenine dinucleotide (FAD; green backbone) are shown as CPK models. **(B)** A close up of the MAO-B active site with *C18* (blue backbone; ball-and-stick model) shows the small-molecule forming a halogen bond (green dotted line) and an H-bond (orange dotted line) with the main chain oxygen atoms of Leu164 and Cys172 (ball-and-stick models with gray backbone), respectively. The binding poses of the coumarin-based inhibitors **(C)** *C18* and **(D)** 7-(3-chlorobenzyloxy)-4-carboxaldehyde-coumarin (*C17* in PDB: 2V60) (Binda et al., 2007) are highly similar with the 3-phenylcoumarin scaffold pose produced by molecular docking. Notably, the coumarin ring is reversed for the established inhibitors in comparison to the docking-based pose of the scaffold. Moreover, the phenyl rings of *C17* and *C18* are attached via ether bonds to the coumarin's C7-position instead of C3-position used with the inhibitors introduced in this study. **(E)** The 2D structure of the 3-phenylcoumarin scaffold indicating the positions of the functional R1-R7 groups.

When inside the neuron, monoamine neurotransmitters such as norepinephrine and dopamine are either recycled or destined for deactivation through oxidative deamination (RCH_2_NHR' + H_2_O + O_2_ = RCHO + R'NH_2_ + H_2_O_2_) by monoamine oxidases A (MAO-A; E.C. 1.4.3.4) and B (MAO-B; E.C. 1.4.3.4). These enzymes are integral monotopic proteins that anchor themselves as dimers onto the mitochondrial outer membrane surface by protruding their α-helical C-termini into the lipid bilayer (Figure 1A). Moreover, both subtypes A and B deaminate preferentially their respective substrates to aldehydes: MAO-A catalyzes serotonin, norepinephrine, and to some extent dopamine; and MAO-B catalyzes dopamine, phenethylamine, benzylamine and to a lesser extent norepinephrine (Shih et al., 1999; Edmondson et al., 2005; Gaweska and Fitzpatrick, 2011).

The MAO-B, which is the target of this study, is connected to neurodegenerative disorders such as Alzheimer's disease but also mental disorders such as schizophrenia, anorexia nervosa, depression and attention deficit disorder. In all of these conditions, the involvement of MAO-B in the metabolism of dopamine and other amines is in a key role (Youdim et al., 2006; Carradori and Silvestri, 2015). For instance, due to gliosis associated with Parkinson's disease, increased levels of MAO-B speed up degradation of dopamine in the motor neurons. MAO-B inhibitors decrease the degradation and boost dopamine concentration in the synapse. Thus, instead of introducing more dopamine, the neurotransmitter levels are elevated by inhibiting MAO-B. As a result, MAO-B inhibitors such as selegiline are used in treatment of Parkinson's disease, moreover, their neuroprotective effects can benefit Alzheimer's disease patients (Youdim et al., 2006). Due to these hepatotoxic effects of irreversibly binding MAO inhibitors, reversible inhibitors such as moclobemide were developed (Youdim et al., 2006; Finberg and Rabey, 2016). The MAO inhibitors can exhibit selectivity toward MAO-A (moclobemide) or MAO-B (pargyline, selegiline) or be non-selective (phenelzine, tranylcypromine). The selectivity, which can be lost in high dosages, is important for avoiding MAO-A inhibition related cheese effect (Youdim et al., 2006; Finberg and Rabey, 2016).

A vast amount of different types of MAO inhibitors are described in the literature and for example the ChEMBL database lists inhibition data for thousands of compounds. The specific problem in the development of MAO-specific ligands is that the promising compounds have potential to become active on other amine oxidases such as vascular adhesion protein 1 (Nurminen et al., 2010, 2011). Here, the aim was to probe the MAO-B activity and selectivity effects of different substitutions on the coumarin core by focusing, especially, on the 3-phenylcoumarin (or 3-arylcoumarin). Notably, there exist two X-ray crystal structures with structurally related coumarin analogs in which 3-chlorobenzyloxy groups are attached at the C7-position (Figures 1B–D). The studied set of 3-phenylcoumarin derivatives with different R1-R7 groups (Figure 1E) introduced in this study make an important addition to the earlier studies in which the potential of coumarin core, including 61 3-phenylcoumarin derivatives (Matos et al., 2009b, 2010, 2011a,b; Santana et al., 2010; Serra et al., 2012; Viña et al., 2012a,b), to block MAO-A and MAO-B has been explored (Borges et al., 2005; Catto et al., 2006; Matos et al., 2009a, 2010, 2011a; Serra et al., 2012; Ferino et al., 2013; Joao Matos et al., 2013; Patil et al., 2013). The compounds were designed using virtual combinatorial chemistry or rationally *de novo* and binding were probed via molecular docking prior to synthesis or *in vitro* testing.

**Figure 2:**
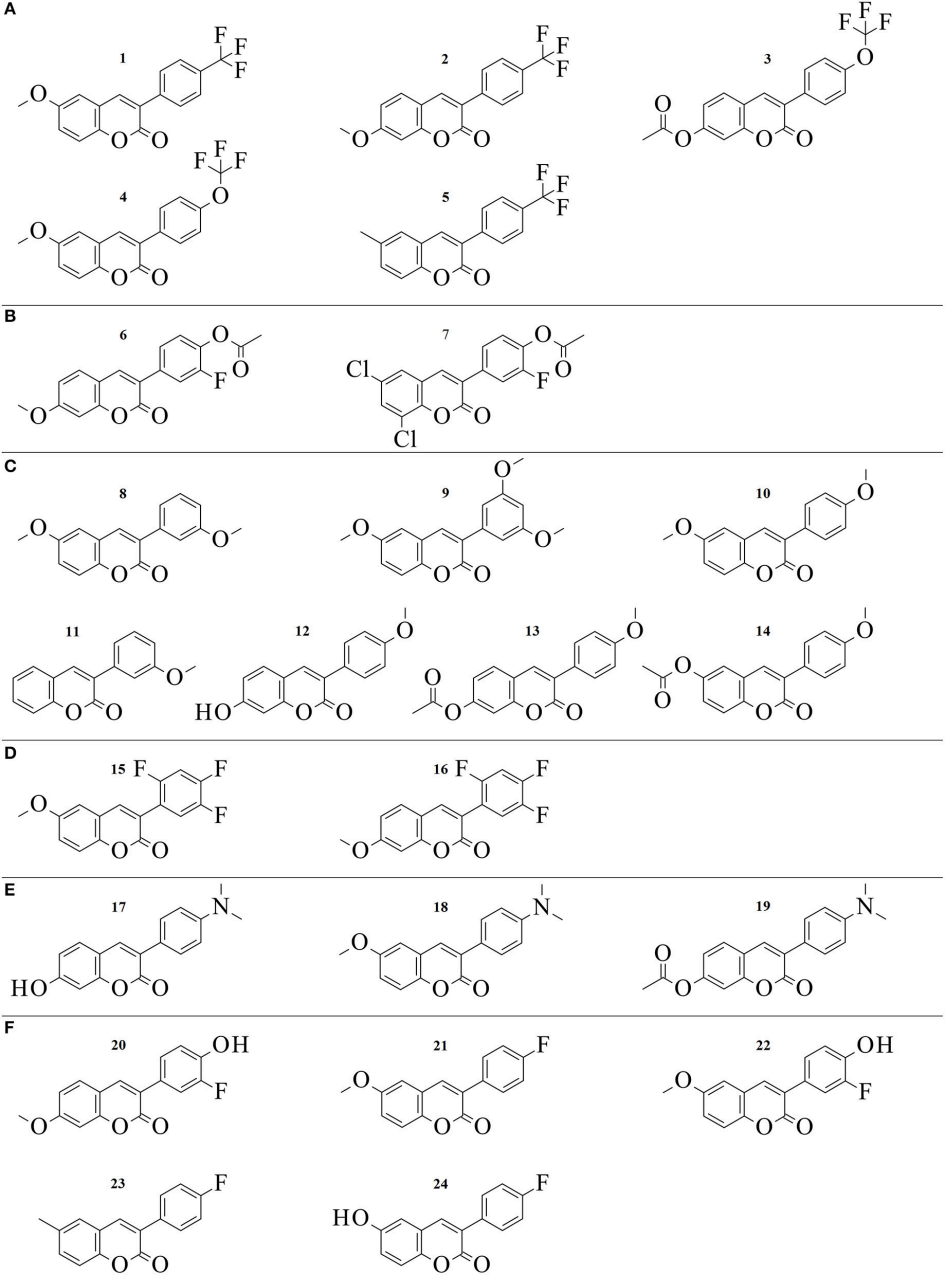
2D structures of the 24 3-phenylcoumarin derivatives producing at least 70% MAO-B inhibition. The compounds are grouped to **(A–F)** groups based on the chemical similarity of the R1-R7 substituents (Figure 1E). See Table 1 for the detailed activity data.

Initially, 52 derivatives of the 3-phenylcoumarin core were synthesized and tested here for the first time for MAO-B inhibition using a specifically tailored spectrophotometric assay (Supplementary Table S1) (Holt et al., 1997). Next, 24 of the derivatives (Figure 2, Table 1), producing >70% inhibition at 10 μM, were selected for further analysis. These derivatives inhibited MAO-B at a ~100 nM to ~1 μM range, while the most potent derivative **1** produces ~50–60 nM inhibition (Table 1, Figure 2). Finally, the potency of the derivatives for inhibiting estrogen receptor (ER), 17-β-hydroxysteroid dehydrogenase 1 (HSD1), aromatase (CYP19A1), and cytochrome P450 1A2 (CYP1A2), the topics of both our prior (Niinivehmas et al., 2016) and ongoing studies, was also considered. A docking-based structure-activity relationship (SAR) analysis (Figure 2) was performed with all of the synthetized 3-phenylcoumarins focusing mainly on the 24 most potent compounds.

**Table 1:** The activity data on the 24 most potent 3-phenylcoumarin derivatives.

**Group**	**ID**	**MAO-B inhibition IC_50_ nM**	**QPlogPo/w**	**MAO-B inhibition % (10 μM)**	**MAO-A inhibition % (100 μM)**	**ER inhibition % (10 μM)**	**HSD1 inhibition % (1 μM)**	**CYP1A2 inhibition IC_50_ μM**
Control	c	61^(1)^	2.43^(1)^	102.00^(1)^	100.89^(2)^	106.60^(3)^	N/A	N/A
A	01	56	4.08	99.53	0.00	N/A	0	124.00
	02	138	4.11	99.58	0.00	N/A	1	N/A
	03	141	3.33	100.44	22.03	N/A	0	280.00
	04	317	4.22	101.96	0.00	N/A	0	7.00
	05	343	4.35	105.33	0.00	1.08	0	171.00
B	06	189	2.47	99.92	0.00	N/A	21	N/A
	07	888	3.36	91.01	0.00	N/A	0	46.00
C	08	231	3.11	111.93	0.00	0	0	2.30
	09	255	3.21	80.21	0.00	N/A	0	84.00
	10	400	3.15	97.57	10.14	N/A	0	15.00
	11	798	3.06	90.33	0.00	0.29	4	1.60
	12	955	2.49	85.89	24.57	91.34	3	170.00
	13	1946	2.41	85.89	2.48	N/A	0	570.00
	14	8476	2.34	75.75	N/A	N/A	1	87.51
D	15	292	3.73	87.16	0.00	0	12	3.00
	16	1433	3.71	77.63	N/A	8.80	33	4.50
E	17	384	2.80	90.14	4.74	N/A	5	35.00
	18	617	3.49	93.86	0.00	0	1	17.00
	19	866	2.79	85.41	0.00	N/A	15	370.00
F	20	391	2.71	100.82	0.00	86.10	46	30.00
	21	433	3.32	88.77	0.00	0	0	3.00
	22	831	2.73	94.86	0.00	55.38	54	1.50
	23	902	3.58	83.49	0.00	0	11	3.00
	24	1058	2.61	89.10	14.18	0	20	3.00

*N/A = not available. Controls: ^(1)^pargyline, ^(2)^clorgyline, ^(3)^kit control. The compounds are grouped (A–F) based on the chemical similarity of the R1–R7 substituents (Figure 1E)*.

In short, this study explores thoroughly the pharmacological potential of 3-phenylcoumarin (Figure 1E) for blocking the MAO-B activity (Table 1, Supplementary Table S1) and, furthermore, explains the basis of the inhibitory effect on the atom level.

## Materials and methods

### Virtual combinatorial chemistry

The 3-phenylcoumarin was chosen as the scaffold of interest for building new MAO-B-specific inhibitors (see section The Alignment of the 3-Phenylcoumarin Scaffold at the Active Site). The analogs were designed using virtual combinatorial chemistry or virtual synthesis. In the initial stages, methoxy group was included at the R1 or R2 position (Figure 1E) in the coumarin core due to its predicted favorability at the active site. The R4-R7 substituents of the 3-phenyl ring (Figure 1E) were designed by combining phenylacetic acid with either 6-methoxycoumarin or 7-methoxycoumarin. The preliminary combinatorial compound library was generated using MAESTRO version 9.3 CombiGlide (CombiGlide, version 2.8, Schrödinger, LLC, New York, NY, USA) and Combinatorial Screening module. The compounds were docked with GLIDE and scored using GlideScore. Some of these derivatives with promising potency and selectivity profile in this study (**8**, **10**, **25**, **37**) were eventually synthesized, albeit using different chemistry (see section Chemical Procedure), and tested *in vitro*. Majority of the final derivatives were designed *de novo* after performing the initial docking simulations with the virtual synthesis products.

### Chemical procedure

All reactions were carried out using commercial materials (Sigma-Aldrich, Mannheim, Germany) and reagents without further purification unless otherwise noted. Reaction mixtures were heated by the CEM Discover microwave apparatus. All reactions were monitored by thin layer chromatography (TLC) on silica gel plates. ^1^H NMR and ^13^C NMR data was recorded on a Bruker Avance 400 MHz spectrometer or Bruker Avance III 300 MHz spectrometer. Chemical shifts are expressed in parts per million values (ppm) and are designated as s (singlet), br s (broad singlet), d (doublet), dd (double doublet), and t (triplet). Coupling constants (*J*) are expressed as values in hertz (Hz). The mass spectra were recorded using Micromass LCT ESI-TOF equipment. Elemental analyses were done with Elementar Vario EL III elemental analyzer. The coumarin derivatives were synthesized using Perkin-Oglialor condensation reaction. The method was developed from the earlier published procedures and transferred to microwave reactor and it was published earlier by authors (Niinivehmas et al., 2016).

A typical procedure: A mixture of salicylaldehyde derivative (2 mmol) and phenyl acetic acid derivative (2.1 mmol), acetic acid anhydride (0.6 ml), and triethylamine (0.36 ml) were placed in a microwave reactor tube and this mixture was heated at 100–170°C with microwave apparatus (100–200 W) for 10–20 min. After cooling, 2 ml of 10% NaHCO_3_ solution was added and the precipitate was filtered, dried and recrystallized from EtOH/H_2_O or acetone/H_2_O mixture. The acetyl group(s) were removed by treating the compound with 2 M MeOH/NaOH(aq) (1:1) solution for 30–60 min at r.t. The solution was acidified with 2 M HCl(aq,) and the precipitate was filtered and recrystallized if needed.

Based on the elemental analysis and/or ^1^H-NMR the purity of compounds was >95%.

**6-methoxy-3-(4-(trifluoromethyl)phenyl)-2H-chromen-2-one (1)**. Yield: 76%; ^1^H-NMR (400 MHz, CDCl_3_) δ: 3.86 (s, 3H, CH_3_O-), 6.99 (s, 1H, H-5), 7.14 (d, 1H, *J*^3^ = 7.7 Hz, H-7), 7.29 (d, *J*^3^ = 8.9 Hz, H-8), 7.69 (d, 2H, *J*^3^ = 7.9 Hz, H-2', H-6'), 7.58 (m, 3H, H-4, H-3', H-5'); ^13^C-NMR (100.6 MHz, CDCl_3_) δ: 55.99, 110.24, 117.73, 119.78, 120.02, 125.51 (q, *J*^C−F^ = 4 Hz), 127.37, 129.05, 130.85 (q, *J*^C−F^ = 32 Hz), 138.41, 140.88, 148.33, 156.44, 160.42. HRMS(ESI): calc. for C_17_H_11_F_3_O_3_Na_1_ 343.0558, found 343.0574; elemental anal. for C_17_H_11_F_3_O_3_, calc. C% 63.76, H% 3.46, found C% 63.25, H% 3.51.

**Scheme 1:**
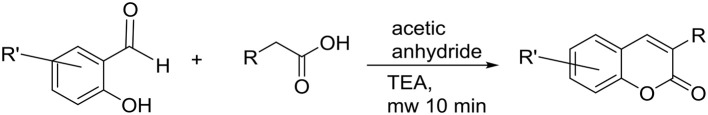
The synthesis of 3-phenylcoumarin analogs.

**6-methoxy-3-(4-(trifluoromethyl)phenyl)-2H-chromen-2-one (2)**. Yield: 80%; ^1^H-NMR (300 MHz, d^6^-DSMO) δ: 3.88 (s, 3H, CH_3_O-), 6.99 (s, 1H, *J*^3^ = 8.7 Hz, *J*^4^ = 2.4 Hz, H-6), 7.03 (d, 1H, *J*^4^ = 2.4 Hz, H-7), 7.71 (d, *J*^3^ = 8.6 Hz, H-8), 7.79 (d, 2H, *J*^3^ = 8.3 Hz, H-2', H-6'), 7.93 (d, 2H, H-3', H-5'), 8.32 (s, 1H, H-4); ^13^C-NMR (75.5 MHz, d^6^-DMSO) δ: 55.97, 100.25, 112.80, 121.57, 122.38, 120.02, 124.97 (q, *J*^C−F^ = 4 Hz), 128.29 (q, *J*^C−F^ = 32 Hz), 128.97, 129.99, 139.01, 142.10, 155.09, 159.62, 162.82. HRMS(ESI) calc for C_17_H_11_F_3_O_3_Na_1_ [M + Na]^+^: 343.05525, found 343.05610.

**2-oxo-3-(4-(trifluoromethoxy)phenyl)-2H-chromen-7-yl acetate (3)**. (Dobelmann-Mara et al., 2017) Yield: 54%; %; ^1^H-NMR (400 MHz, d^6^-DMSO) δ: 2.27 (s, 3H, CH_3_C(O)O-), 7.20 (dd, 1H, *J*^3^ = Hz, *J*^4^ = Hz, H-6), 7.33 (d, 1H, *J*^4^ = Hz, H-8), 7.47 (d, 2H, *J*^3^ = Hz, H-3', H-5'), 7.81 (d, 1H, *J*^3^ = 8.4 Hz, H-5), 8.32 (s, 1H, H-4); ^13^C-NMR (100 MHz, d^6^-DMSO) δ: 20.86 109.74, 117.23, 118.88, 120.75, 124.84, 129.42, 129.60, 130.52, 133.85, 140.73, 148.35, 152.90, 153.55, 159.40, 168.78; HRMS(ESI) calc. for C_18_H_11_F_3_O_5_Na_1_ [M + Na]^+^ 387.0457, found 387.0481.

**6-methoxy-3-(4-(trifluoromethoxy)phenyl)-2H-chromen-2-one (4)**. Yield: 52%; ^1^H-NMR (400 MHz, CDCl_3_) δ: 3.86 (s, 3H, CH_3_O-), 6.98 (d, 1H, *J*^4^ = 3 Hz, H-5), 7.12 (dd, *J*^3^ = 9.1 Hz, *J*^4^ = 3 Hz, H-7), 7.27-7.30 (m, 3H, H-8, H-3', H-5'), 7.74 (d, 2H, *J*^3^ = 8.9 Hz, H-2', H-6'); 7.77 (s, 1H, H-4); ^13^C-NMR (100 MHz, CDCl_3_) δ: 55.99, 110.16, 117.70, 119.68, 119.92, 120.97, 127.41, 130.26, 133.51, 140.20, 148.21, 149.67, 156.41, 160.65. HRMS(ESI) calc for C_17_H_11_F_3_O_4_Na_1_ [M + Na]^+^: 359.05071, found 359.05260. elemental anal. for C_17_H_11_F_3_O_4_·0.5H_2_O, calc. C% 59.14, H% 3.50, found C% 58.99, H% 3.25.

**6-methyl-3-(4-(trifluoromethyl)phenyl)-2H-chromen-2-one (5)**. Yield: 54%; ^1^H-NMR (400 MHz, CDCl_3_) δ: 7.27 (d, 1H, *J*^4^ = 2.2 Hz, H-5), 7.35-7.38 (m, 2H, H-7, H-8), 7.70 (d, *J*^3^ = 8.2 Hz, H-2', H-6'), 7.82 (m, 3H, H-4, H-3', H-5'); ^13^C-NMR (100 MHz, CDCl_3_) δ: 20.92, 116.46, 119.22, 122.80, 125.53 (q, *J*^C−F^ = 4 Hz), 126.98, 128.07, 129.05, 130.80 (q, *J*^C−F^ = 33 Hz), 133.29, 134.62, 138.50, 141.08, 152.04 160.53; HRMS(ESI) calc for C_17_H_12_Cl_2_O_4_Na_1_ [M + Na]^+^: 373.0005, found 372.9998.

**2-fluoro-4-(7-methoxy-2-oxo-2H-chromen-3-yl)phenyl acetate (6)**. Yield 75%; ^1^H-NMR (400 MHz, d^6^-DMSO) δ: 2.35 (s, 3H, CH_3_C(O)O-Ph), 3.88 (s, 3H, CH_3_O-Ph), 6.99 (dd, 1H, *J*^3^ = 8.6 Hz, *J*^4^ = 2.4 Hz, H-6), 7.05 (d, 1H, *J*^4^ = 2.4 Hz, H-8), 7.37 (dd, *J*^3^ = 9.3 Hz, *J*^H−F^ = 8.3 Hz, H-5'), 7.62 (ddd, 1H, *J*^3^ = 8.5 Hz, *J*^4^ = 2.1 Hz, *J*^H−F^ = 0.8 Hz, H-6'), 7.68 (d, *J* = 8.6 Hz, 1H, H-5), 7.74 (dd, *J*^H−F^ = 12.1 Hz, *J*^4^ = 2.0 Hz, H-3'), 8.31 (s, 1H, H-4); ^13^C-NMR (100 MHz, d^6^-DMSO) δ: 20.19, 55.97, 100.25, 112.79, 116.35 (d, *J*^C−F^ = 20.3 Hz), 121.02, 121.03, 123.83, 124.79 (d, *J*^C−F^ = 3.2 Hz), 129.86, 134.24 (d, *J*^C−F^ = 7.7 Hz), 137.20 (d, *J*^C−F^ = 13.1 Hz), 141.55, 153.00 (*J*^C−F^ = 246.1 Hz), 154.92, 159.65, 162.69, 168.19. HRMS(ESI) calc for C_18_H_13_F_1_O_5_Na_1_ [M + Na]^+^: 351.06447, found 351.06240; elemental anal. for C_18_H_13_F_1_O_5_ C% 65.85, H% 3.99, found C% 65.28 H% 4.02.

**4-(6,8-dichloro-2-oxo-2H-chromen-3-yl)-2-fluorophenyl acetate (7)**. Yield 58%; ^1^H-NMR (400 MHz, d^6^-DMSO) δ: 2.36 (s, 3H, CH_3_C(O)O-), 7.43 (dd, *J*^3^ = 9.3 Hz, *J*^H−F^ = 8.3 Hz, H-5'), 7.67 (ddd, 1H, *J*^3^ = 8.4 Hz, *J*^4^ = 2.1 Hz, *J*^H−F^ = 0.8 Hz, H-6'), 7.74 (dd, *J*^H−F^ = 11.8 Hz, *J*^4^ = 2.0 Hz, H-3'), 7.84 (d, 1H, *J*^4^ = 2.4 Hz, H-7), 7.97 (d, 1H, *J*^4^ = 2.4 Hz, H-5), 8.32 (s, 1H, H-4); ^13^C-NMR (100 MHz, d^6^-DMSO) δ: 20.72, 117.23 (d, *J*^C−F^ = 21 Hz), 121.13, 122.17, 124.65, 125.74 (d, *J*^C−F^ = 3.3 Hz), 127.29, 128.80, 131.47, 133.70 (d, *J*^C−F^ = 7.7 Hz), 138.50 (d, *J*^C−F^ = 12.9 Hz), 140.12, 147.94, 152.30, 154.75. 158.73; HRMS(ESI): calc. for C_17_H_9_Cl_2_F_1_O_4_Na_1_ [M + Na]^+^: 388.9760, found 388. 9762.

**6-methoxy-3-(3-methoxyphenyl)-2H-chromen-2-one (8)**. Yield 78%; ^1^H-NMR (400 MHz, CDCl_3_) δ: 3.85 (s, 3H, CH_3_O-Ph), 3.86 (s, 3H, CH_3_O-Ph), 6.93-6.97 (m, 2H, H-4', H-5), 7.10 (dd, 1H, *J*^3^ = 9.0, Hz, *J*^4^ = 1.9 Hz, H-7), 7.25-7.29 (m, 3H, H-8, H-2', H-6'), 7.35 (t, 1H, *J*^3^ = 8.2 Hz, H-5'), 7.76 (s, 1H, H-4); ^13^C-NMR (100 MHz, CDCl_3_) δ: 55.69, 56.18, 110.28, 114.57, 114.88, 117.78, 119.55, 120.28, 121.26, 128.80, 129.79, 136.43, 140.13, 148.34, 156.47, 159.88, 160.91; HRMS(ESI): calc. for C_17_H_14_O_4_Na_1_ [M + Na]^+^: 305.07898, found 305.07950; elemental anal. for C_14_H_14_O_4_ calc. C% 72.33, H% 5.00, found C% 72.41, H% 4.88.

**3-(3,5-dimethoxyphenyl)-6-methoxy-2H-chromen-2-one (9)**. (Vilar et al., 2006) Yield 59%; ^1^H-NMR (400 MHz, d^6^-DMSO) δ: 3.79 (s, 6H, CH_3_O-Ph), 3.82 (s, 3H, CH_3_O-Ph), 6.56 (t, 1H, *J*^4^ = 2.3 Hz, H-4'), 6.89 (d, 2H, *J*^4^ = 2.3 Hz, H-2', H-6'), 7.20 (dd, 1H, *J*^3^ = 9.0 Hz, *J*^4^ = 3.0 Hz, H-7), 7.31 (d, 1H, *J*^4^ = 3.0 Hz, H-5), 7.36 (d, 1H, *J*^3^ = 9.0 Hz, H-8), 8.23 (s, 1H, H-4); ^13^C-NMR (100 MHz, d^6^-DMSO) δ: 55.30, 55.66, 100.48, 106.71, 110.69, 116.90, 119.36, 119.78, 126.75, 136.44, 140.66, 147.33, 155.62, 159.53, 160.16; HRMS(ESI): calc. for C_18_H_16_O_5_Na_1_ [M + Na]^+^: 335.08954, found 305.09010; elemental anal. for C_14_H_14_O_4_ calc. C% 69.22, H% 5.16, found C% 68.80, H% 5.14.

**6-methoxy-3-(4-methoxyphenyl)-2H-chromen-2-one (10)**. (Prendergast, 2001; Ferino et al., 2013) Yield 79%; ^1^H-NMR (400 MHz, CDCl_3_) δ: 3.847 (s, 3H, CH_3_O-Ph), 3.852 (s, 3H, CH_3_O-Ph), 6.95-6.98 (m, 3H, H-5, H-3', H-5'), 7.07 (dd, 1H, *J*^3^ = 9.0 Hz, *J*^4^ = 2.9 Hz, H-7), 7.27 (d, 1H, *J*^4^ = 8.8 Hz, H-5), 7.66 (d, 2H, *J*^3^ = 8.9 Hz, H-2', H-6'), 7.70 (s, 1H, H-4); ^13^C-NMR (100 MHz, CDCl_3_) δ:55.69, 56.16, 110.11, 114.23, 117.69, 119.05, 120.51, 127.47, 128.49, 130.18, 138.63, 148.11, 156.44, 160.49, 161.24; HRMS(ESI): calc. for C_17_H_14_O_4_Na_1_ [M + Na]^+^: 305.07898, found 305.07910; elemental anal. for C_17_H_14_O_4_ calc. C% 72.33, H% 5.00, found C% 72.34, H% 4.86.

**3-(3-methoxyphenyl)-2H-chromen-2-one (11)**. (Kirkiacharian et al., 1999) Yield 81%; ^1^H-NMR (400 MHz, CDCl_3_) δ: 3.86 (s, 3H, CH_3_O-Ph), 6.95 (ddd, 1H, *J*^3^ = 8.2 Hz, *J*^4^ = 2.3 Hz, *J*^4^ = 2.5 Hz, H-4'), 7.26-7.37 (m, 5H, H-6, H-8, H-2', H-5', H-6'), 7.51-7.53 (m, 2H, H-5, H-7), 7.81 (s, 1H, H-4); ^13^C-NMR (100 MHz, CDCl_3_) δ: 55.69, 114.56, 114.86, 116.76, 119.94, 121.24, 124.81, 128.26, 128.49, 129.80, 131.76, 136.35, 140.28, 153.85, 159.88, 160.76; HRMS(ESI): calc. for C_16_H_12_O_3_Na_1_ [M + Na]^+^: 275.06841, found 275.06540; elemental anal. for C_16_H_12_O_3_ calc. C% 76.18, H% 4.79, found C% 75.94, H% 4.67.

**7-hydroxy-3-(4-methoxyphenyl)-2H-chromen-2-one (12)**. (Prendergast, 2001) Yield 81%; ^1^H-NMR (400 MHz, d^6^-DMSO) δ: 3.79 (s, 3H, CH_3_O-Ph), 6.74 (s, 1H, H-8), 6.81 (d, 1H, *J*^3^ = 8.5 Hz, H-6), 6.99 (d, 2H, *J*^3^ = 8.3 Hz, H-3', H-5'), 7.57 (d, 1H, *J*^3^ = 8.4 Hz, H-5), 7.65 (d, 2H, *J*^3^ = 8.3 Hz, H-2, H-6'), 8.08 (s, 1H, H-4), 10.54 (s, 1H, HO-Ph); ^13^C-NMR (100 MHz, d^6^-DMSO) δ: 55.18, 101.66, 112.10, 113.29, 113.61, 121.84, 127.30, 129.48, 129.70, 139.73, 154.63, 159.14, 160.20, 160.88; HRMS(ESI): calc. for C_16_H_12_O_4_Na_1_ [M + Na]^+^: 291.06333, found 291.06160.

**3-(4-methoxyphenyl)-2-oxo-2H-chromen-7-yl acetate (13)**. (Bhandri et al., 1949) Yield 67%; ^1^H-NMR (400 MHz, d^6^-DMSO) δ: 7.02 (d, 2H, *J*^3^ = 7.8 Hz, H-3', H-5'), 7.17 (d, 1H, *J*^3^ = 8.3 Hz, H-6), 7.29 (d, 1H, H-8), 7.69 (d, 2H, *J*^3^ = 7.8 Hz, H-2', H-6'), 7.79 (d, 1H, *J*^3^ = 8.2 Hz, H-5), 8.19 (s, 1H, H-4); ^13^C-NMR (100 MHz, d^6^-DMSO) δ: 20.85, 55.22, 109.57, 113.68, 117.49, 118.68, 125.69, 126.72, 129.19, 129.75, 138.62, 152.36, 153.15, 159.59, 168.81; HRMS (ESI): Calc for C_18_H_14_O_5_Na_1_ [M + Na]^+^: 333.07389, found 333.07220. Elemental analysis for C_18_H_14_O_5_ calc C% 69.67 H% 4.55, found C% 69.58 H% 4.52.

**3-(4-methoxyphenyl)-2-oxo-2H-chromen-6-yl acetate (14)**. Yield 34%; 1H-NMR (300 MHz, d6-DMSO) δ: 2.31 (s, 3H, CH3C(O)O-), 3.81 (s, 3H, CH3O-), 7.03 (d, 2H, J3 = 8.7 Hz, H-3', H-5'), 7.37 (dd, 1H, J3 = 8.9 Hz, J4 = 2.5 Hz, H-7), 7.47 (d, 1H, J3 = 8.9 Hz, H-8), 7.54 (d, 1H, J4 = 2.5 Hz, H-5), 7.70 (d, 2H, J3 = 8.7 Hz, H-2', H-6'), 8.15 (s, 1H, H-5); 13C-NMR (75 MHz, d6-DMSO) δ: 20.73, 55.21, 113.68, 116.83, 120.07, 120.53, 125.00, 126.61, 127.03, 129.84, 138.30, 146.39, 150.18, 159.64, 159.71, 169.22. HRMS (ESI): Calc for C18H14O5 [M + H]+: 311.0914, found 311.0908.

**6-methoxy-3-(2,4,5-trifluorophenyl)-2H-chromen-2-one (15)**. Yield 80%; ^1^H-NMR (400 MHz, d^6^-DMSO) δ: 3.81 (s, 3H, CH_3_O-Ph), 7.26 (dd, 1H, *J*^3^ = 9.0 Hz, *J*^4^ = 3.0 Hz, H-7), 7.31 (d, 1H, *J*^4^ = 3.0 Hz, H-5), 7.41 (d, 1H, *J*^3^ = 9.0 Hz, H-8), 7.64-7.77 (m, 2H, H-2', H-6'), 8.18 (s, 1H, H-4); ^13^C-NMR (100 MHz, d^6^-DMSO) δ: 55.73, 106.31 (dd, *J*^C−F^ = 21 Hz, *J*^C−F^ = 22 Hz), 110.90, 117.25, 119.12, 119.39, 119.55, 120.07, 120.91, 143.74, 145.70 (d, *J*^C−F^ = 242 Hz), 147.64, 149.34 (*J*^C−F^ = 252 Hz), 155.13 (*J*^C−F^ = 248 Hz), 155.79, 158.78. HRMS (ESI): Calc for C_16_H_9_F_3_O_3_Na_1_ [M + Na]^+^: 329.04015, found 329.04090. Elemental analysis for C_16_H_9_F_3_O_3_: calc C% 62.75 H% 2.96, found C% 62.62 H% 3.15.

**7-methoxy-3-(2,4,5-trifluorophenyl)-2H-chromen-2-one (16)**. Yield 85 %; ^1^H-NMR (300 MHz, d^6^-DMSO) δ: 3.88 (s, 3H, CH_3_O-Ph), 7.00 (dd, 1H, *J*^3^ = 8.6 Hz, *J*^4^ = 2.4 Hz, H-6), 7.06 (d, 1H, *J*^4^ = 2.3 Hz, H-8), 7.61-7.6 (m, 3H, H-5, H-2', H-6'), 8.17 (s, 1H, H-4); ^13^C-NMR (75.5 MHz, d^6^-DMSO) δ: 56.02, 100.49, 106.21 (dd, *J*^C−F^ = 21 Hz, *J*^C−F^ = 21 Hz), 112.24, 112.85, 116.85, 119.30, 119.57, 129.95, 144.06, 145.67 (d, *J*^C−F^ = 242 Hz), 148.93 (d, *J*^C−F^ = 250) Hz, 155.10 (d, *J*^C−F^ = 245 Hz), 155.22, 158.89, 162.98; HRMS (ESI): Calc for C_16_H_9_F_3_O_3_Na_1_ [M + Na]^+^: 329.04015, found 329.03980.

**3-(4-(dimethylamino)phenyl)-7-hydroxy-2H-chromen-2-one (17)**. (Kirkiacharian et al., 2003) In the first step 7-acetoxy-3-(4-(dimethylamino)phenyl)-2*H*-chromen-2one was obtained. Yield: 70%; ^1^H-NMR (400 MHz, d^6^-DMSO) δ: 2.31 (s, 3H, CH_3_C(O)O-Ph), 2.95 (s, 6H, (CH_3_)_2_N-Ph), 6.77 (d, *J*^3^ = 9.0 Hz, 2H, H-2', H-6'), 7.14 (dd, *J*^3^ = 8.4 Hz, *J*^4^ = 2.2 Hz, 1H, H-5), 7.26 (d, *J*^4^ = 2.2 Hz, 1H, H-8), 7.63 (d, *J*^3^ = 9.0 Hz, 2H, H-3', H-5') 7.76 (d, *J*^3^ = 8.5 Hz, 1H, H-5), 8.11 (s, 1H, H-4); ^13^C-NMR (100.6 MHz, d^6^-DMSO) δ: 20.85, 39.84, 109.44, 111.58, 117.76, 118.57, 121.57, 126.00, 128.82, 129.11, 136.46, 150.45, 151.90, 152.77, 159.74, 168.85. In the second step 7-hydroxy-3-(4-(dimethylamino)phenyl)-2*H*-chromen-2one was obtained. Yield: 85% yellow solid; ^1^H-NMR (400 MHz, d^6^-DMSO) δ: 2.94 (s, 6H, (CH_3_)_2_N-), 6.72 (d, *J*^4^ = 2.3 Hz, 1H, H-8), 6.75 (d, *J*^3^ = 9.0 Hz, 2H, H-2', H-6'), 6.79 (dd, *J*^3^ = 8.4 Hz, *J*^4^ = 2.3 Hz, 1H, H-5),), 7.55 (d, *J*^3^ = 8.5 Hz, 1H, H-5), 7.58 (d, *J*^3^ = 9.0 Hz, 2H, H-3', H-5'), 7.99 (s, 1H, H-4); ^13^C-NMR (100.6 MHz, d^6^-DMSO) δ: 39.92, 101.59, 112.33, 113.16, 122.30, 122.32, 129.34, 137.83, 150.07, 154.27, 160.30, 160.41; HRMS (ESI): Calc for C_17_H_15_N_1_O_3_Na_1_ [M + Na]^+^: 304.09496, found 304.09480; elemental anal. for C_17_H_15_N_1_O_3_, calc. C% 72.58, H% 5.37, N% 4.98, found C% 72.45, H% 5.40, N% 5.15.

**3-(4-(dimethylamino)phenyl)-6-methoxy-2H-chromen-2-one (18)**. Yield 55%; ^1^H-NMR (400 MHz, d^6^-DMSO) δ: 2.96 (s, 6H, (CH_3_)_2_N-Ph), 3.81 (s, 3H, CH_3_O-Ph), 6.77 (d, 2H, *J*^3^ = Hz, H-3', H-5'), 7.14 (dd, 1H, *J*^3^ = 3.0 Hz, *J*^4^ = 9.0 Hz, H-7), 7.28 (d, 1H, *J*^4^ = 3.0 Hz, H-5), 7.33 (d, 1H, *J*^3^ = 9.0 Hz, H-8), 7.63 (d, 2H, *J*^3^ = 9.0 Hz, H-2', H-6'), 8.06 (s, 1H, H-4); ^13^C-NMR (100.6 MHz, d^6^-DMSO) δ: 39.93, 110.27, 111.59, 116.68, 118.16, 120.35, 121.73, 126.96, 129.15, 136.79, 146.79, 150.46, 155.59, 160.06; HRMS (ESI): Calc for C_18_H_17_N_1_O_3_Na_1_ [M + Na]^+^: 318.11061, found 318.11050; elemental anal. for C_18_H_17_N_1_O_3_, calc. C% 73.20, H% 5.80, N% 4.74, found C% 72.75, H% 5.83, N% 4.45.

**3-(4-(dimethylamino)phenyl)-2-oxo-2H-chromen-7-yl acetate (19)**. Yield 70%; ^1^H-NMR (400 MHz, d^6^-DMSO) δ: 2.31 (s, 3H, CH_3_C(O)O-Ph), 2.95 (s, 6H, (CH_3_)_2_N-Ph), 6.77 (d, 2H, *J*^3^ = 9.0 Hz, H-3', H-5'), 7.14 (dd, 1H, *J*^3^ = 8.4 Hz, *J*^4^ = 2.2 Hz, H-6), 7.26 (d, 1H, *J*^4^ = 2.2 Hz, H-8), 7.63 (d, 2H, *J*^3^ = 9.0 Hz, H-2', H-6'), 7.76 (d, 1H, *J*^3^ = 8.5 Hz, H-5), 8.11 (s, 1H, H-4; ^13^C-NMR (100 MHz, d^6^-DMSO) δ: 20.86, 39.84, 109.44, 111.58, 117.76, 118.57, 121.58, 126.00, 128.82, 129.11 136.46, 150.45, 151.90, 152.77, 159.74, 168.85; HRMS (ESI): Calc for C_19_H_17_N_1_O_4_Na_1_ [M + Na]^+^: 346.10553, found 346.10640.

**3-(3-fluoro-4-hydroxyphenyl)-7-methoxy-2*H*-chromen-2-one (20)**. In the first step 2-fluoro-4-(7-methoxy-2-oxo-2H-chromen-3-yl)phenyl acetate was obtained. Yield 75%; ^1^H-NMR (400 MHz, d^6^-DMSO) δ: 2.35 (s, 3H, CH_3_C(O)O-Ph), 3.88 (s, 3H, CH_3_O-Ph), 6.99 (dd, 1H, *J*^3^ = 8.6 Hz, *J*^4^ = 2.4 Hz, H-6), 7.05 (d, 1H, *J*^4^ = 2.4 Hz, H-8), 7.37 (t, 1H, *J* = 8.3Hz, H-6'), 7.62 (d, *J* = 8.5 Hz, 1H, H-5'), 7.68 (d, *J* = 8.6 Hz, 1H, H-5), 7.74 (dd, *J*^H−F^ = 12.1 Hz, *J*^4^ = 2.0 Hz, H-3'), 8.31 (s, 1H, H-4); ^13^C-NMR (100 MHz, d^6^-DMSO) δ: 20.19, 55.97, 100.25, 112.79, 116.35 (d, *J*^C−F^ = 20.3 Hz), 121.02, 121.03, 123.83, 124.79 (d, *J*^C−F^ = 3.2 Hz), 129.86, 134.24 (d, *J*^C−F^ = 7.7 Hz), 137.20 (d, *J*^C−F^ = 13.1 Hz), 141.55, 153.00 (*J*^C−F^ = 246 Hz), 154.92, 159.65, 162.69, 168.19. In the second step 3-(3-fluoro-4-hydroxyphenyl)-7-methoxy-2*H*-chromen-2-one was obtained. Yield 70%; ^1^H-NMR (400 MHz, d^6^-DMSO) δ: 3.87 (s, 3H, CH_3_O-Ph), 6.96-7.03 (m, 3H, H-6, H-8, H-5'), 7.41 (d, 1H, *J*^3^ = 8.4, H-6'), 7.57 (dd, 1H, *J*^H−F^ = 13.1 Hz, *J*^4^ = 2.2 Hz (H-H), 1H, H-2'), 7.66 (d, 1H, *J*^3^ = 8.4, H-5), 8.18 (s, 1H, H-4), 10.09 (s, 1H, Ph-OH). ^13^C-NMR (75.5 MHz, d^6^-DMSO) δ: 55.91, 100.16, 112.61, 113.04, 115.95 (d, *J*^C−F^ = 20 Hz), 117.37 (d, *J*^C−F^ = 3.3 Hz), 121.78 (*J*^C−F^ = 2.0 Hz), 124.54 (d, *J*^C−F^ = 3.0 Hz), 126.08 (d, *J*^C−F^ = 7.0 Hz), 129.49, 139.62, 145.0 (*J*^C−F^ = 13 Hz), 150.46 (d, *J*^C−F^ = 240 Hz), 154.52, 159.87, 162.19; HRMS (ESI): Calc for C_16_H_11_F_1_O_4_Na_1_ [M + Na]^+^: 309.0539, found 309.0553.

**3-(4-fluorophenyl)-6-methoxy-2H-chromen-2-one (21)**. Yield 58%; ^1^H-NMR (400 MHz, d^6^-acetone) δ: 3.87 (s, 3H, CH_3_O-Ph), 7.19-7.33 (m, 5H, H-5, H-7, H-8, H-3', H-5'), 7.83 (dd, 2H, *J*^HF^ = 5.4 Hz, *J*^H−H^ = 9.0 Hz, H-2', H6'), 8.12 (s, 1H, H-4); ^13^C-NMR (100 MHz, d^6^-acetone) δ: 56.17, 111.34, 115.84 (d, *J*^C−F^ = 22 Hz), 117.85, 120.04, 121.04, 127.79, 131.62 (d, *J*^C−F^ = 8 Hz), 132.41 (d, *J*^C−F^ = 3 Hz), 140.82, 148.82, 157.13, 160.64, 163.72 (d, *J*^C−F^ = 247 Hz); HRMS (ESI): Calc for C_16_H_11_F_1_O_3_Na_1_ [M + Na]^+^: 293.05899, found 293.05850; elemental anal. for C_16_H_11_F_1_O_3_, calc C% 71.11, H% 4.10, found C% 71.10, H% 4.10.

**3-(3-fluoro-4-hydroxyphenyl)-6-methoxy-2*H*-chromen-2-one (22)**. In the first step 2-fluoro-4-(6-methoxy-2-oxo-2H-chromen-3-yl)phenyl acetate was obtained. Yield 66%; ^1^H-NMR (400 MHz, d^6^-DMSO) δ: 2.33 (s, 3H, CH_3_C(O)O-Ph), 3.82 (s, 3H, (CH_3_O-Ph), 7.23 (dd, 1H, *J*^3^ = 9.0 Hz, *J*^4^ = 3.0 Hz, H-7), 7.30 (d, 1H, *J*^4^ = 3.0 Hz, H-5), 7.35 (d, 1H, *J*^3^ = 9.2 Hz, H-8), 7.61 (d, 1H, *J*^3^ = 8.5 Hz, H-5'), 7.75 (dd, 1H, *J*^H−F^ = 12.0 Hz, *J*^4^ = 1.7 Hz (H-H), 1H, H-3'), 8.30 (s, 1H, H-4); ^13^C-NMR (100.6 MHz, d^6^-DMSO) δ: 20.22, 55.69, 110.83, 116.67, 117.02, 119.66, 123.96, 125.10, 135.96, 141.18, 147.44, 151.78, 154.23, 155.70, 159.53, 168.21. In the second step 3-(3-fluoro-4-hydroxyphenyl)-6-methoxy-2*H*-chromen-2-one was obtained. Yield 71%; ^1^H-NMR (400 MHz, d^6^-DMSO) δ: 3.81 (s, 3H, (CH_3_O-Ph), 7.02 (dd, 1H, *J*^3^ = 9.2 Hz, H-6'), 7.18 (dd, 1H, *J*^3^ = 9.0 Hz, *J*^4^ = 3.0 Hz, H-7), 7.28 (d, 1H, *J*^4^ = 2.9 Hz, H-5), 7.42 (d, 1H, *J*^3^ = 8.4 Hz, H-5'), 7.57 (dd, 1H, *J*^H−F^ = 13.0 Hz, *J*^4^ = 2.2 Hz (H-H), 1H, H-2'), 8.17 (s, 1H, H-4), 10.19 (s, 1H, Ph-OH); ^13^C-NMR (100.6 MHz, d^6^-DMSO) δ: 55.66, 110.59, 116.67, 117.02, 119.66, 123.96, 125.10, 135.96, 141.18, 147.44, 151.78, 154.23, 155.70, 159.53, 168.21. HRMS (ESI): Calc for C_16_H_11_F_1_O_4_Na_1_ [M + Na]^+^: 309.0539, found 309.0521.

**3-(4-fluorophenyl)-6-methyl-2H-chromen-2-one (23)**. (Chauhan et al., 2016) Yield 74%; ^1^H-NMR (400 MHz, d^6^-DMSO) δ: 2.38 (s, 3H, CH_3_-Ph), 7.27-7.35 (m, 3H, H-3', H-5', H-8), 7.43 (dd, 1H, *J*_3_ = 8.5 Hz, *J*_4_ = 2.1 Hz, H-7), 7.55 (d, 1H, *J*_4_ = 1.4 Hz, H-5), 7.77 (dd, 2H, *J*^HF^ = 5.7 Hz, *J*^H−H^ = 9.0 Hz, H-2', H6'), 8.18 (s, 1H, H-4); ^13^C-NMR (100.6 MHz, d^6^-DMSO) δ: 20.26, 115.11 (d, *J*^H−F^ = 21.5 Hz), 115.64, 119.16, 125.76, 128.20, 130.70 (d, *J*^H−F^ = 8.4 Hz), 131.10 (d, *J*^H−F^ = 3.2 Hz), 132,61, 133.80, 140.48, 151.10, 159.82, 162.17 (d, *J*^H−F^ = 245 Hz); HRMS (ESI): Calc for C_16_H_11_F_1_O_2_Na_1_ [M + Na]^+^: 277.06408, found 277.06390; Elemental anal. for C_16_H_11_F_1_O_2_, calc C% 75.58, H% 4.36, found C% 75.42, H% 4.33.

**3-(4-fluorophenyl)-6-hydroxy-2H-chromen-2-one (24)**. In the first step 3-(4-fluorophenyl)-2-oxo-2H-chromen-6-yl acetate was obtained and used as such for the next step. In the second step 3-(4-fluorophenyl)-6-hydroxy-2H-chromen-2-one was obtained. Yield 65%; ^1^H-NMR (300 MHz, d^6^-DMSO) δ: 7.04 (dd, 1H, *J*^3^ = 8.8 Hz, *J*^4^ = 2.9 Hz, H-7), 7.09 (d, 1H, *J*^4^ = 2.8 Hz, H-5), 7.24-7.29 (m, 3H, H-3', H-5', H-8), 7.75 (dd, 2H, *J*^HF^ = 5.6 Hz, *J*^H−H^ = 8.9 Hz, H-2', H6'), 8.13 (s, 1H, H-4), 9.72 (s, 1H, HO-Ph); ^13^C-NMR (75.5 MHz, d^6^-DMSO) δ: 112.561, 115.03 (d, *J*^H−F^ = 21.5 Hz), 116.71, 119.78, 119.93, 125.80, 130.70 (d, *J*^H−F^ = 8.2 Hz), 131.18 (d, *J*^H−F^ = 3.2 Hz), 140.50, 146.35, 159.92, 162.17 (d, *J*^H−F^ = 246 Hz); HRMS (ESI): Calc for C_15_H_9_F_1_O_3_Na_1_ [M + Na]^+^: 279.04334, found 279.0444.

### Monoamine oxidase A and B

Both monoamine oxidase A (MAO-A) and B (MAO-B) protein and the reagents for the chromogenic solution of vanillic acid (4-hydroxy-3-methoxylbenzoic acid, 97% purity), 4-aminoantipyrine (reagent grade), horseradish peroxidase and the substrate tyramine hydrochloride (minimum 99% purity) as well as the potassium phosphate buffer, which was prepared using potassium phosphate dibasic trihydrate (≥99% ReagentPlus™) and potassium phosphate monobasic (minimum 98% purity, molecular biology tested), were purchased from Sigma-Aldrich (St. Louis, MO, USA) for the spectrophotometric assay.

The protocol for continuous spectrophotometric assay (Holt et al., 1997) was followed in the activity measurements. The assay was performed in 0.2 M potassium phosphate buffer pH 7.6 on 96-well plates (Nunc™ 96F microwell plate without a lid, Nunc A/S, Roskilde, DK) in 200 μl total volume. The chromogenic solution containing 1 mM vanillic acid, 500 μM 4-aminoantipyrine and 8 U/ml horseradish peroxidase in 0.2 M potassium phosphate buffer pH 7.6 was mixed anew for each measurement. 5 mM tyramine solution was used as the substrate. In order to determine the activity of both MAO-B and MAO-A, concentration series as duplicates were prepared. The protein was combined with the chromogenic solution and incubated 30 min at 37°C. The background signal was measured using multilabel reader (Victor^TM^ X4, 2030 Multilabel Reader, PerkinElmer, Waltham, MA, USA) at A_490_ before reaching the total 200 μl volume by adding 20 μl of tyramine to final concentration of 0.5 mM on the plate. As a result, the final concentration of the chromogenic solution on the plate was 250 μM vanillic acid, 125 μM 4-aminoantipyrine and 2 U/ml horseradish peroxide. After adding the substrate, the plates were measured 300 times every 15 s using 1 s exposure time. The device was set to 37°C for the duration of the experiment.

Based on the activity measurement, suitable concentrations were chosen for both MAO-B and MAO-A to be used in the inhibition studies (Supplementary Figures S1, S2, and S5, Table 1, Supplementary Table S1). The experiment conditions should produce absorbance change of ~0.35 (Holt et al., 1997). With MAO-B, this was reached using 10 μl (equals 50 μg of protein with enzymatic activity 3.2 units per well) of the protein and running the experiment for 2 h (Supplementary Figures S1, S2, and S5, Table 1, Supplementary Table S1). MAO-A was significantly more active, providing absorbance change of >0.5 with 5 μl (equals 25 μg of protein with enzymatic activity 1.05 units per well) of protein and, consequently, the reaction maximum was reached already in 30 min (Supplementary Figure S5, Table 1, Supplementary Table S1). Thus, a wide panel of coumarin derivatives was analyzed at 10 μM (Table 1, Supplementary Table S1) and those 3-phenylcoumarin derivatives producing >70% inhibition were selected for further analysis (Table 1, Figure 2). The selected 24 candidates were measured as duplicates on a dilution series ranging from 50 μM to 1 nM, and based on the normalized measurement results, IC_50_ values were calculated (Table 1). The same wide panel of coumarin derivatives was additionally used to analyze the MAO-A inhibition at 100 μM (Table 1, Supplementary Table S1).

GRAPHPAD PRISM 5.03 (GraphPad Software Inc., CA, USA) was used to normalize the spectrophotometric assay data where the maximal signal was reached at the lowest concentration of 10^−8^ or 10^−9^ depending on the sample and the starting concentration of 5·10^−5^ acted as the lowest point of signal. The measured data was then fitted on a curve using non-linear regression with the equation for log[inhibitor] vs. response. The IC50 values were therefore determined based on the curve fit. The fitted curves are shown on –log scale in Supplementary Figures S1, S2.

### 17-β-hydroxysteroid dehydrogenase 1

Inhibition of the 17-β-hydroxysteroid dehydrogenase 1 (HSD1) was determined by HPLC using recombinant human HSD1 proteins, produced in Sf9-insect cells, as described earlier (Messinger et al., 2009). The assay was performed in a final volume of 0.2 ml buffer (20mM KH2PO4, 1mM EDTA, pH 7.4) containing 0.1 mg/ml protein, 1 mM cofactor NADPH, 30 nM substrate estrone or estradiol, 800,000 cpm/ml of tritium labeled estrone ([3H]-E1) or estradiol ([3H]-E2) and inhibitor concentrations in the range of 0.1–5 mM. Triplicate samples were incubated for 25 min at RT. The reaction was stopped by addition of 20 ml 10% trichloroacetic acid per sample. After incubation the substrate and the product of enzymatic conversion [3H]-E1 and [3H]-E2, were separated and quantified by HPLC (Alliance 2790, Waters) connected to an online -counter (Packard Flow Scintillation Analyzer). The ratio of [3H]-E1 converted to [3H]-E2, or *vice versa*, determines the sample conversion percentage. Inhibition efficiencies were calculated by comparing the conversion percentages of the samples including inhibitors with those of conversion controls (without inhibitors).

### Aromatase

Aromatase (CYP19A1) activity was measured as described previously (Pasanen, 1985) by using human placental microsomes and 50 nM [3H]-androstenedione as a substrate and inhibitor concentrations in the range of 60–1,000 nM. Aromatase activities were measured as released [3H]-H_2_O in Optiphase Hisafe 2 scintillation liquid (Perkin Elmer, USA) with a Wallac 1450 MicroBeta Trilux scintillation counter (Perkin Elmer, USA). As a positive control for aromatase inhibition, 1 μM finrozole (generous gift from Olavi Pelkonen, University of Oulu, Finland) was used.

### Cytochrome P450 1A2

Inhibition of CYP1A2 activity was determined with commercial heterologously expressed human CYP1A2 enzyme (Corning Inc., Corning, NY, USA) as described earlier (Korhonen et al., 2005). The metabolic activity was not in the scope of this particular study. The assay was adapted to the 96-well plate format. In each well, a 150 μL incubation volume contained 100 mM Tris-HCl buffer (pH 7.4), 4.2 mM MgCl2,1 μM 7-ethoxyresorufin, 0.5 pmol of cDNA expressed CYP1A2, 0-40 mM inhibitor, and a NADPH-generating system. All inhibitors were dissolved in ethanol, and the final concentration of ethanol was 2% in all incubations. The reaction was initiated by adding the NADPH-regenerating system after a 10 min preincubation at 37°C, and after a 20 min incubation, the reaction was terminated by the addition of 110 μL of 80% acetonitrile/20% 0.5 M Tris base. The formed fluorescence was measured with a Victor2 plate counter (Perkin-Elmer Life Sciences Wallac, Turku, Finland) at 570 nm excitation and 616 nm emission.

### Estrogen receptor

The pIC50 values for the derivatives (Table 1, Supplementary Table S1) were measured with green PolarScreen™ ER Alpha Competitor Assay (Life Technologies, CA, The United States of America) kit, following the manufacturer protocol as previously described (Niinivehmas et al., 2016). The final concentration of the compounds ranged from 0.0007 to 10 000 nM in the dilution series which were performed as duplicates. The molecules were combined with 25 nM ERα and 4.5 nM fluormone in the assay buffer and placed on black low volume 384-well assay plate with NBS surface (Corning, NY, The United States of America). After mixing the assay plate, it was incubated for 2 h in RT. The fluorescence polarization was measured using excitation wave length 485 and emission wave length 535 with bandwidths of 25/20 nm on a 2104 EnVision® Multilabel Plate Reader which had EnVision Workstation version 1.7 (PerkinElmer, MA, The United States of America).

### Computational methods

**Figure 3:**
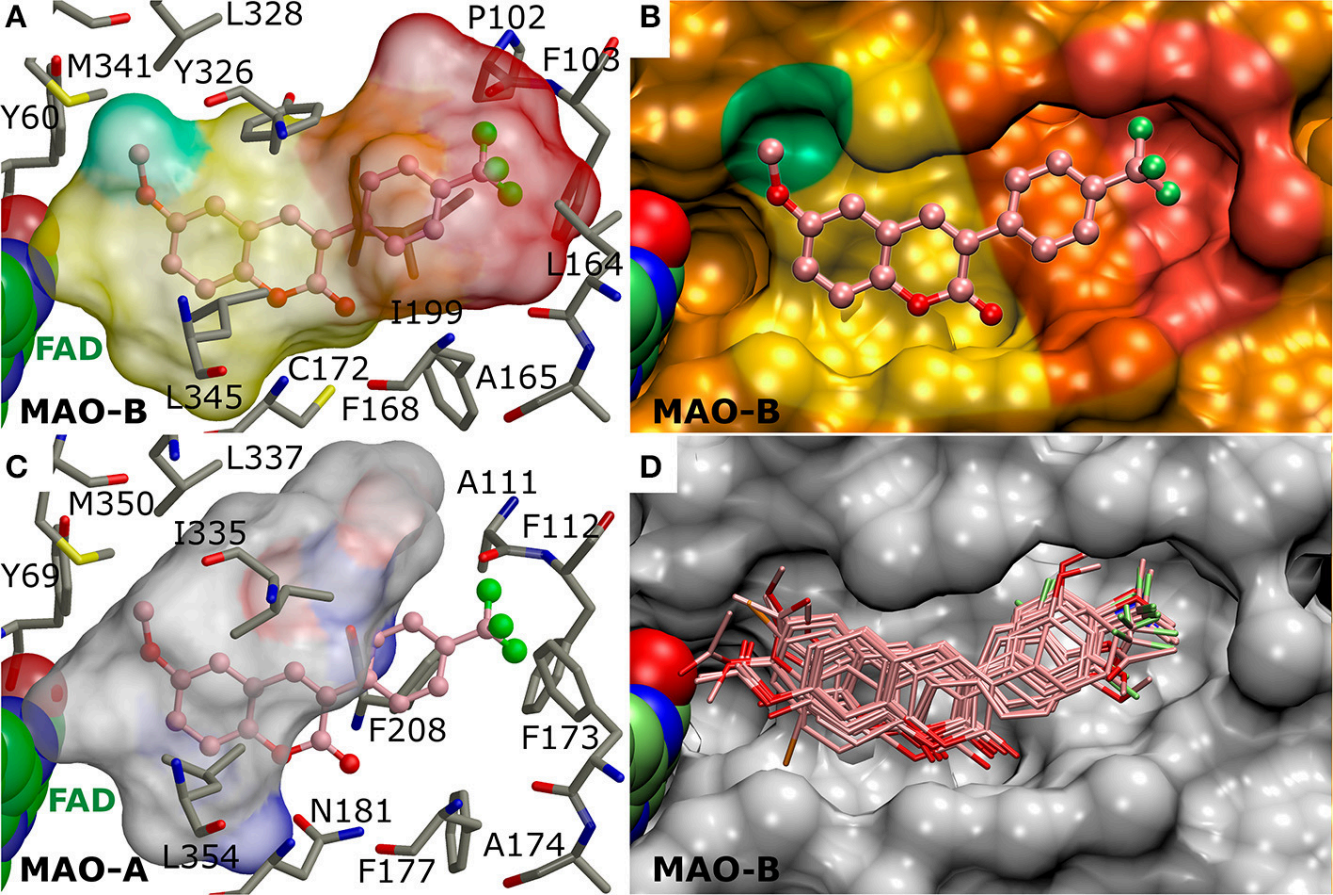
The active site of monoamine oxidase B with docked 3-phenylcoumarin derivatives. **(A)** A negative image of the MAO-B active site shown as a transparent surface indicates the space available for inhibitor binding with docked derivative **1** (ball-and-stick model; Figure 2). **(B)** A cross section, showing half of the active site, displays the contours (opaque surface) that roughly match the inhibitor shape and conformation. The colored sectors highlight specific sections of the cavity dedicated to different aspects of the 3-phenylcoumarin derivative binding: 3-phenyl ring (orange), the R4-R7 groups of the 3-phenyl ring (red), coumarin ring (yellow), the hydrophobic niche occupied by the R1/R2-groups of the coumarin ring (green). **(C)** A negative image of the MAO-A active site shows that only two residue changes (Ile199 → Phe208; Leu164 → Phe173) are enough to prevent 3-phenylcoumarin analog binding. **(D)** The docked poses of the 23 most potent 3-phenylcoumarin derivatives show what space is collectively occupied by the new inhibitors. See Figure 1 for details.

**Figure 4:**
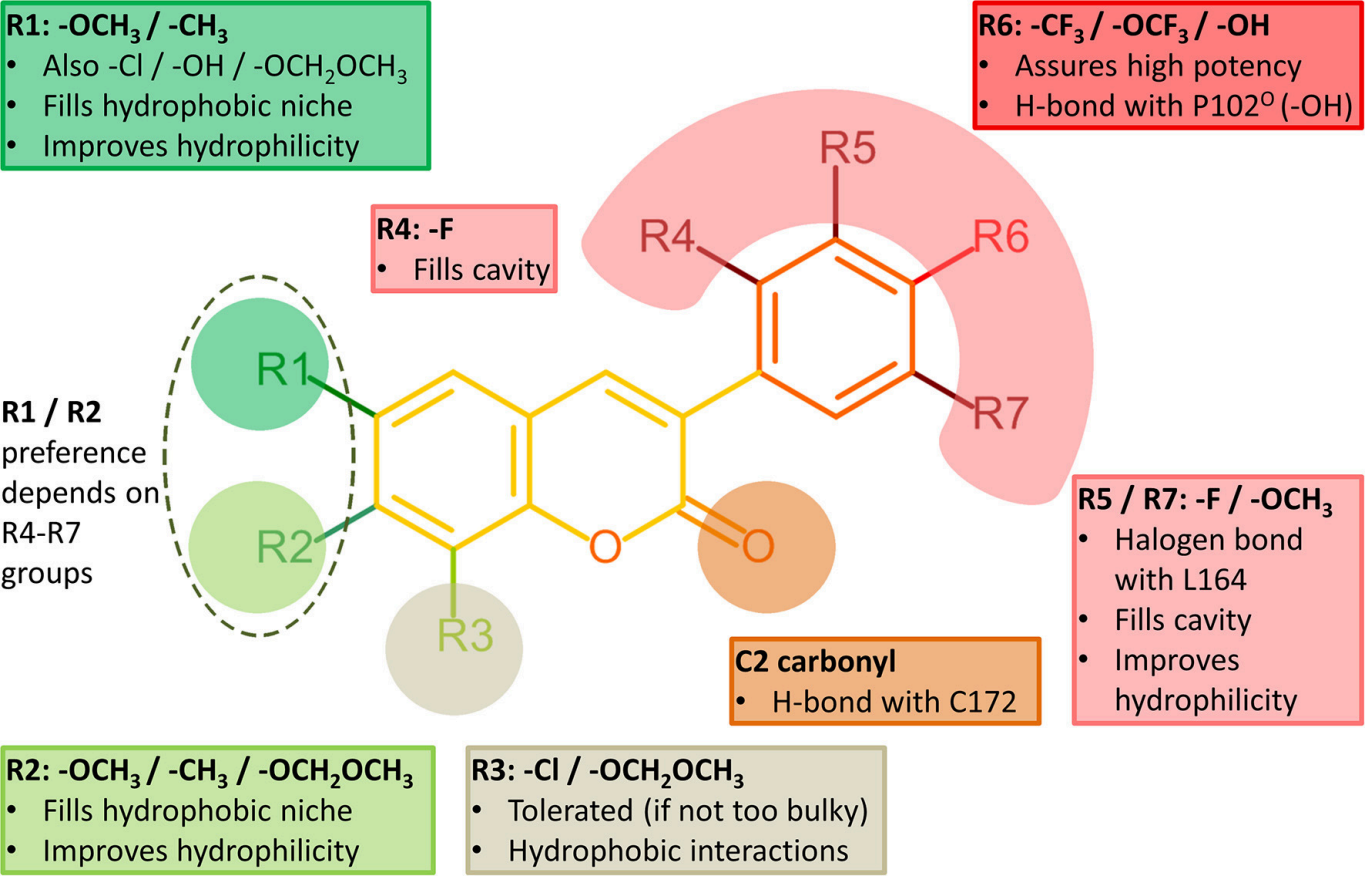
Structure-activity relationship (SAR) analysis of the 3-phenylcoumarin derivatives.

**Figure 5:**
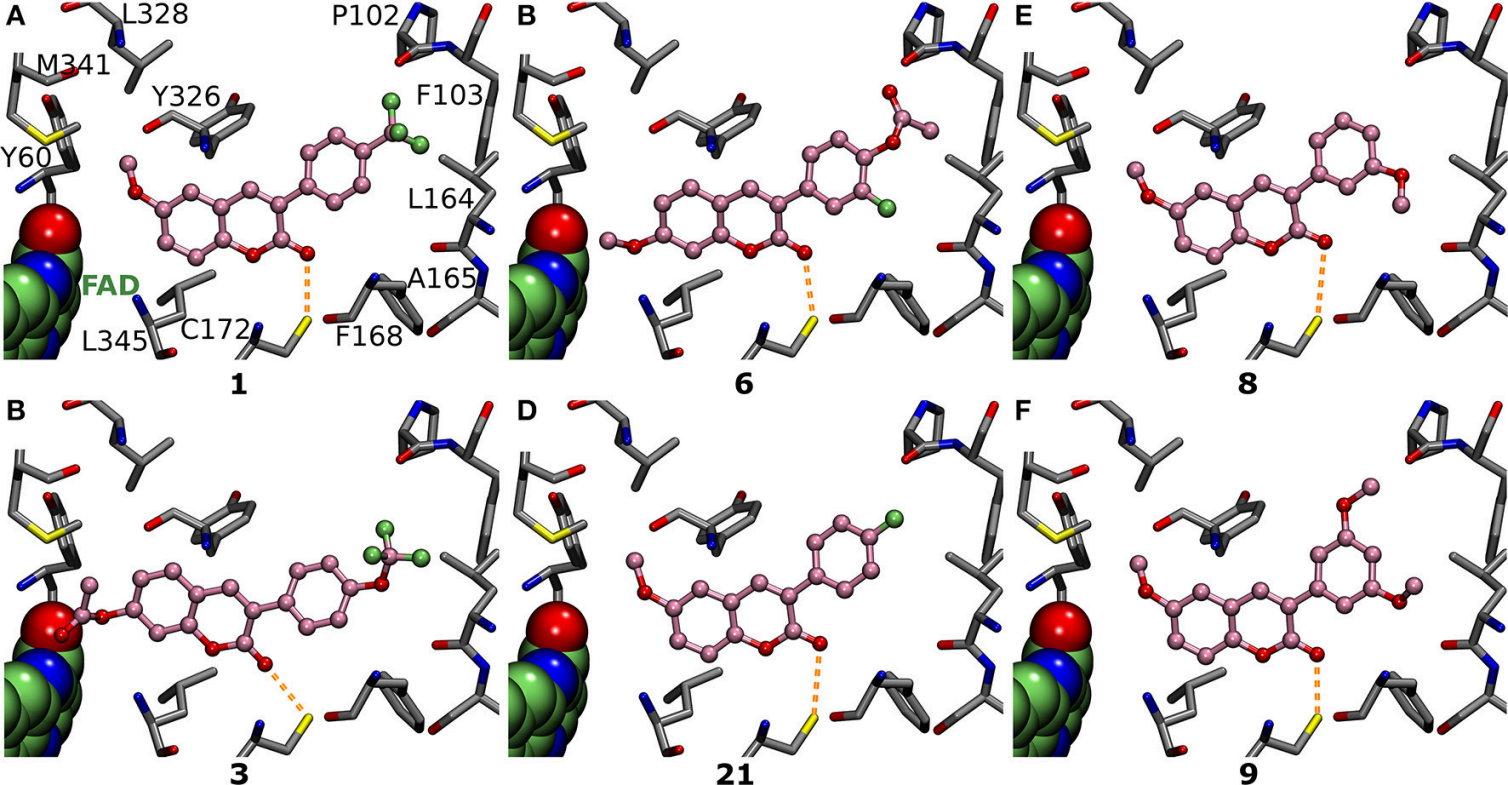
The vital role of R4-R7 substituents of the 3-phenyl ring for the inhibition. Focusing on the 3-phenyl ring, the derivatives (ball-and-stick models with pink backbone) elicit strong MAO-B inhibition via **(A)** R6-trifluoromethyl (**1**; Figure 2; IC_50_ of 56 nM; Table 1), **(B)** R6-trifluoromethoxy (**3**; Figure 2; IC_50_ of 141 nM; Table 1), **(C)** R6-acetoxy and R7-fluorine (**6**; Figure 2; IC_50_ of 189 nM; Table 1), **(D)** R6-fluorine (**21**; Figure 2; IC_50_ of 433 nM; Table 1), **(E)** R7-methoxy (**8**; Figure 2; IC_50_ of 231 nM; Table 1), and **(F)** R5- and R7-methoxy (**9**; Figure 2; IC_50_ of 255 nM; Table 1) groups. See Figure 1 for further details.

The small-molecule ligand structures were drawn in 3D and their tautomeric states at pH 7.4 were built using LIGPREP module in MAESTRO 2016-3 (Schrödinger, LLC, New York, NY, USA, 2016). The derivatives were docked to the X-ray crystal structure of MAO-B (PDB: 2V60) (Binda et al., 2007) with PLANTS 1.2 (Korb et al., 2009) using 10 Å radius and the C8 atom of inhibitor *C18* (PDB: 2V60) was used as the center. The R1-methoxy group rotamers of compounds **1**, **8**, **9**, **21**, **15**, **18**, and **22** were manually adjusted to indicate how the groups exploit the small hydrophobic niche in the cavity (green sector in Figures 3A,B). The 2D structures of the 3-phenylcoumarin scaffold and the 24 most potent inhibitor derivatives shown in Figures 1E, **2** were drawn with BIOVIA DRAW 2016 (Dassault Systèmes, San Diego, CA, USA, 2016). Figures 1A–D, 3–5 were prepared using BODIL (Lehtonen et al., 2004) and VMD 1.9.2 (Humphrey et al., 1996). The negative images of the MAO-B and MAO-A binding cavities shown in Figure 3A and C were outlined using PANTHER (Niinivehmas et al., 2011, 2015) and visualized with BODIL, MOLSCRIPT (Kraulis, 1991), and RASTER3D (Merritt and Murphy, 1994).

## Results and discussion

### Spectrophotometric activity measurements for monoamine oxidase B

All of the 52 derivatives were docked, synthetized and tested experimentally. Those 24 compounds that provided IC_50_ values below 10 μM were tested more thoroughly (Table 1). The fact that 24 of the synthesized derivatives with a wide variety of different R1-R7 groups (Figure 2) passed the 70% threshold indicates that the 3-phenylcoumarin is indeed a highly suitable scaffold for building MAO-B inhibitors. Notably, eight of these tested derivatives (**3**, **9**–**13**, **17**, and **23** in Figure 2) had been synthesized previously (Bhandri et al., 1949; Kirkiacharian et al., 1999, 2003; Prendergast, 2001; Vilar et al., 2006; Ferino et al., 2013; Chauhan et al., 2016; Dobelmann-Mara et al., 2017), however, this is the first time they are tested for MAO-B activity. The novel derivative **1** is the most potent inhibitor of the analog set with the IC_50_ value of 56 nM (Figure 2, Table 1); meanwhile, the rest of the tested derivatives are evenly distributed within a range of 0.1–10 μM (Figure 2, Table 1).

By focusing solely on the R1-R7 constituents of the derivatives (Figures 1E, 2) and the activity data (Table 1) it is possible to outline trends that determine which functional groups, positions or their combinations establish and weaken or improve the MAO-B inhibition.

Although the R1 and R2 groups in the coumarin ring are not necessarily required for establishing MAO-B inhibition (see **11**; Figure 2, Supplementary Figure S3F; Table 1), the activity measurements indicate that adding a methoxy, hydroxyl, acetoxy, methyl or even halogen group(s) into the ring can facilitate strong inhibition (Table 1). As a rule of thumb, introducing R1-methoxy group produces strong MAO-B inhibition (e.g., **1**; Figure 2; Table 1). In contrast, inserting for example a bulky R3 substituent such as acetoxy group weakens the inhibition considerably (26, 35, 47; Supplementary Figure S4; Supplementary Table S1). Whether the R1 or R2 position or any specific functional group in particular is favored depends on the composition of the 3-phenyl ring's R4-R7 constituents.

In fact, the activity data indicates that the R4-R7 substituents are vital for assuring strong MAO-B inhibition and without any 3-phenyl substituents, the activity is lost (41, 50, 52; Supplementary Figure S4, Supplementary Table S1). The most potent inhibitors were **1** (IC_50_ of ~56 nM; Table 1) and **2** (IC_50_ of ~138 nM; Table 1) housing R6-trifluoromethyl, but **3** (IC_50_ of ~141 nM; Table 1) with structurally similar R6-trifluoromethoxy group is almost equally potent. The combination of the R6-acetoxy and R7-fluorine groups in **6** (IC_50_ of ~189 nM) produces relatively strong inhibition. Furthermore, housing just one methoxy group at the R7 position (**8**; IC_50_ of ~230 nM) or two methoxy groups at both R5 and R7 positions (**9**; IC_50_ of ~255 nM) assures < 300 nM inhibition.

The effects of the R4-R7 groups of the 3-phenyl ring and the R1-R3 groups of coumarin ring (Figure 2) for the derivative binding and inhibition are detailed below in a docking-based structure-activity relationship (SAR) analysis.

### The alignment of the 3-phenylcoumarin scaffold at the active site

The 3-phenylcoumarin derivative binding at the MAO-B active site is based on the premise that the coumarin and phenyl ring systems occupy roughly the same 3D space as the equivalent ring systems of the coumarin-based inhibitors co-crystallized with the enzyme (PDB: 2V60, 2V61; Figures 1A–D) (Binda et al., 2007). The fundamental difference between the 3-phenylcoumarin derivatives and those coumarin inhibitors with validated binding poses is that the coumarin alignment is reversed and the phenyl ring is attached to the C3-position instead of the C7-position (Figures 1C,D).

What is more, the “canonical” coumarin ring positioning inside the pocket is somewhat analogous to even simpler double ring constructs such as the indole of inhibitor isatin (PDB: 1OJA) (Binda et al., 2003). In fact, the hydrophobicity of the aromatic coumarin (yellow sector in Figures 3A,B) and 3-phenyl (orange sector in Figures 3A,B) rings is vital for establishing the MAO-B binding and it outweighs all other favorable interactions such as hydrogen or halogen bonding (*via* sigma hole) in importance (Figure 4). Thus, although the docking suggests variability in the coumarin and 3-phenyl ring positioning for the 3-phenylcoumarin derivatives due to different R1-R7 substituents, the hydrophobic interactions of the aromatic rings are highly similar between them (Figure 3D).

It is also noteworthy that the coumarin's C2-carbonyl is not facing the solvent based on the molecular docking simulations (Figure 3D). Paradoxically, this does not matter, because the carbonyl group finds an atypical interaction partner from the thiol group of Cys172 side chain (Figure 4). Although the C2-carbonyl cannot form a full-fledged H-bond with the proton of the thiol group, the hydrophobic environment of the cavity likely enhances this ordinarily weak interaction between the two groups.

### R6-trifluoromethyl packing produces the strongest inhibition

Halogen substituents in the 3-phenyl ring ensure strong MAO-B inhibition (Figure 4). This makes sense with MAO-B, because despite their apparent electronegativity the halogen substituents actually improve the steric packing of small-molecules via persistent van der Waals interactions while also retaining the ability to act as a halogen bond donor. Both of these properties should assist inhibitor binding into the active site that is mostly hydrophobic (Figures 3A,B). Besides, the increased lipophilicity conveyed by the halogen substituents (logP values in Table 1) should assist the 3-phenylcoumarin derivatives in aggregating on the outer mitochondrial membrane on route to the MAO-B active site (Figure 1A).

The most potent derivative **1** (Figure 2, Table 1) has trifluoromethyl group at the R6 position in the 3-phenyl ring. The derivative is relatively flat when bound at the active site and the proximal R6-group cannot flex out of this plane (Figure 5A). The trifluoromethyl of **1** fits very snugly into the hydrophobic end of the cavity (red sector in Figures 3A,B). The high shape complementarity of this cavity part and the R6-trifluoromethyl of **1** is typical for this bulky moiety in drug compounds. Thus, the R6-group alignment of **1** is mostly relying on the collective potency of individually weak van der Waals interactions (Figures 3A,B, 5A).

Replacing the R6-trifluoromethyl of derivative **1** with a trifluoromethoxy in **4** (Figure 2) produces six times lower MAO-B inhibition (Table 1, Supplementary Figure S3B). This happens because the trifluoromethoxy already fills the available space almost optimally (Figures 3A,B, 5A) and elongating the substituent with an ether bond does not improve the fit (Supplementary Figure S3B). In fact, there is no extra wiggle room to fit the trifluoromethoxy (Figures 3A,B), if the 3-phenylcoumarin scaffold would be kept at the “canonical” position (Figures 1C,D). Hence, the coumarin ring of **4** pushes slightly closer to the cofactor. Although the binding site residues can adjust slightly in response to this shift, the realignment or rather misalignment of the scaffold (Supplementary Figure S3B) imposes an energetic cost that is reflected in the MAO-B inhibition (Table 1). In addition, depending on the rotamer pose of the R6-trifluorometoxy, a hydrogen bond could be bridged between a fluorine atom and the Pro102^O^ by a water molecule (not shown).

### The effects of halogenation on the 3-phenyl ring alignment

The chlorine and fluorine substituents of prior coumarin-based inhibitors form halogen bond with the Leu164^O^ based on X-ray crystallography (PDB: 2V60, 2V61; Figures 1A–D; Binda et al., 2007). Accordingly, it is not surprising that those 3-phenylcoumarin derivatives with single halogen substituent at their 3-phenyl rings are also capable of blocking the MAO-B activity (Figure 4, Table 1).

**Figure 6:**
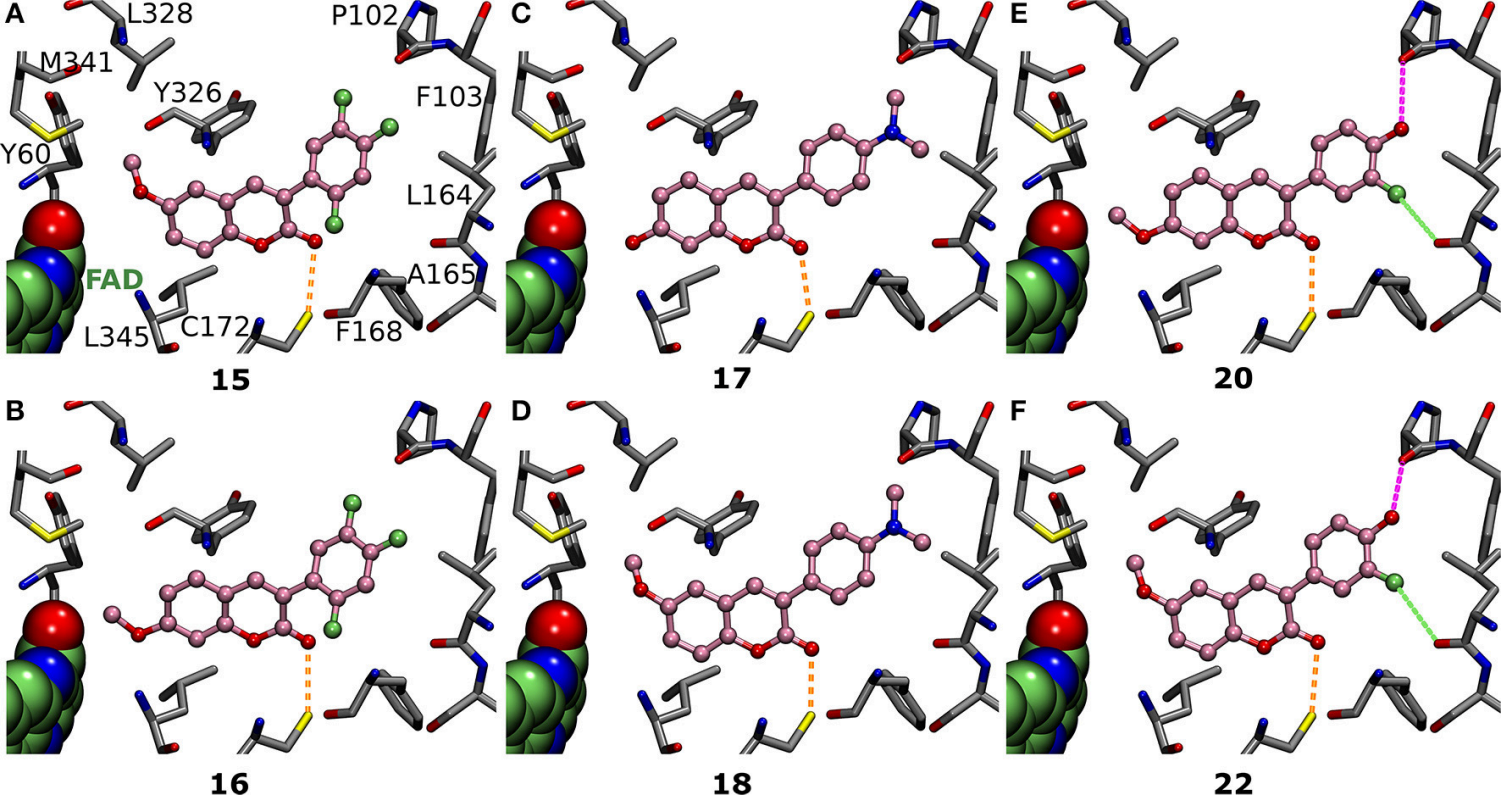
The effects of R1 and R2 substituents are dependent on the 3-phenyl ring substituents. **(A)** Derivatives **15** (Figure 2; IC_50_ of 292 nM; Table 1) and **(B) 16** (Figure 2; IC_50_ of 1433 nM; Table 1) both have fluorine groups at R4, R6, and R7 positions, but switching the coumarin ring's R1-methoxy into the R2 position reduces the inhibition by whopping 1141 nM. In contrast, with **(C) 17** (Figure 2; IC_50_ of 384 nM; Table 1) and **(D) 18** (Figure 2; IC_50_ of 617 nM; Table 1), the R1-methoxy does not elicit as strong inhibition as the R2-hydroxyl due to the overall coumarin ring alignment dictated by the 3-phenyl's R5-dimethylamine. The R1/R2-methoxy switch produces a completely opposite effect for **(E) 20** (Figure 2; IC_50_ of 391 nM; Table 1) and **(F) 22** (Figure 2; IC_50_ of 831 nM; Table 1) than it did for **15** and **16** (panels **A,B**); namely, it lowered the inhibition by 440 nM (Table 1). For further details, see Figures 1, 4.

Although it is known that fluorine is the poorest halogen bond donor (Cavallo et al., 2016), the R7-fluorine groups of **20** and **22** (Figure 2) could form halogen bond with the Leu164° (Figures 6E,F) similarly to the halogens of previously published inhibitors with validated binding modes (Figures 1B–D; Binda et al., 2007). In fact, the R7-halogen groups of **20** and **22** are inserted into the exact same position as the halogen groups of the established inhibitors (Figure 1B vs. Figures 6E,F). The MAO-B inhibition (Table 1) is reinforced further by the R6-hydroxyl group H-bonding with the Pro102^O^ (magenta dotted lines in Figures 6E,F). Because both **20** and **22** are bonding simultaneously with the Leu164^O^ and the Pro102^O^, they elicit equivalent or stronger inhibition than derivatives **21** (Figure 5D), **23** (Supplementary Figure S3K), and **24** (Supplementary Figure S3L) that do not retain either one of these two interactions. Docking suggests that replacing the R6-hydroxyl with an acetoxy group prevents **6** (Figure 2) from forming direct halogen or hydrogen bonds (Figure 5C), but the R6-acetoxy and R7-fluorine could potentially connect via a water bridge with the Pro102^O^ (not shown). Despite this, the hydrophobic packing of the R6-acetoxy in **6** against the hydrophobic residues, mainly Phe103 (Figure 5C), is likely the reason behind doubling the inhibition in comparison to **20** (IC_50_ value of 391 vs. 189 nM; Table 1, Figure 6E).

Introducing fluorine to the R6 position of the 3-phenyl ring in derivatives **21**, **23**, and **24** (Figure 2) produces MAO-B inhibition ranging from 433 to 1,060 nM (Table 1). Due to the overall planarity of the 3-phenylcoumarin scaffold (Figures 1C,D), the R6-fluorine (Figure 5D, Supplementary Figures S3K,L), cannot take on the equivalent site occupied by the halogens of validated coumarin-based inhibitors that form halogen bond with the Leu164° (Figure 1B; Binda et al., 2007). In addition, the R6-fluorine is too limited in size to fill the end of the binding cavity as completely as for example the trifluoromethyl of **1** does (Figures 3A,B, 5A). In addition, the R6-fluorine groups of derivatives **21**, **23**, and **24** (Figure 5D, Supplementary Figure S3K,L) reside within a suitable distance to form a halogen bond with the Pro102^O^ (3.6 Å), however, the available angles seem to rule out actual bonding.

Derivatives **15** and **16** (Figure 2) house three fluorine atoms at their 3-phenyl groups' R4, R6, and R7 positions (Figures 6A,B). In the case of **15** (Figure 6A), these halogen substituents assure an IC_50_ value that is almost 150 nM stronger than what is seen with derivatives housing only a single fluorine moiety at the R6 or R7 position (**21**, **23**, and **24**; Figure 5D, Figure S3K-L, Table 1). This is achieved by filling the hydrophobic cavity end (orange and red sectors in Figure 3) efficiently with the 3-phenyl ring and its fluorine moieties (Figures 6A,B). The fit is better for a 3-phenyl ring with the R5-trifluoromethyl than what is seen with the ring housing three separate fluorine substituents (Figure 5A vs. Figure 6A) and; accordingly, derivative **15** is not as potent MAO-B inhibitor as **1** (IC_50_ 292 vs. 56 nM; Table 1). In addition, depending on the 3-phenyl ring pose, the R4 or R7 fluorine groups could again potentially act as weak halogen bond donors to the Phe168^O^ or the Leu164^O^, respectively (not shown).

### The effects of the methoxy and dimethylamine groups for the 3-phenyl alignment

Derivatives with proximal methoxy groups (Figure 2), especially at the R7 position, assure relatively strong MAO-B inhibition (Figure 4) and produce at best 230 nM inhibition (e.g., **8** in Figure 2, Table 1).

Based on the docking, derivatives **8** and **11** (Figure 2) flip their R7-methoxy groups toward the Leu164° (Figure 5E, Supplementary Figure S3F), which is shielded from a clash with the methoxy group by forming intra-protein H-bond with the Phe168^N^ (not shown). Inserting an extra R5-methoxy into the 3-phenyl of **8** to produce otherwise identical derivative **9** (Figure 2) weakens the inhibition slightly (IC_50_ difference of 23 nM; Table 1), because the added methoxy group is unable to form particularly favorable interactions with the nearby Pro102° (Figure 5F). With derivatives **10** or **13** (Figure 2), the methoxy group is added to the phenyl ring's para position, and due to the planarity of the 3-phenylcoumarin scaffold, there is an energetic penalty for pushing the group toward either side of the cavity end (red sector in Figures 3A,B). Accordingly, to avoid a scaffold misalignment, the R6-methoxy group of **10** (and **13**) points directly toward the side chains of Phe103, Pro104, Trp119, and Ile199 (Supplementary Figures S3E,F), which, in turn, produces roughly 170 nM difference in the IC_50_ values with otherwise identical **8** (Figure 5E, Table 1) in favor of the R7-methoxy position.

A dimethylamine group at the 3-phenyl ring's para position (a.k.a. dimethylaniline; Figure 2) produces moderately strong MAO-B inhibition (Table 1) for derivatives **17** (Figure 2; IC_50_ value of 400 nM), **18** (Figure 2; IC_50_ value of 798 nM), and **19** (Figure 2; IC_50_ value of 955 nM). This is due to the ability of the R6-dimethylamine to fill the cavity end (red sector in Figures 3A,B) similarly to the R6-trifluoromethyl of **1** (Figures 5A,B vs. Figures 6C,D, Supplementary Figure S3J). The downside is that the bulkier R6-substituent cannot form halogen or hydrogen bonds with water or residues nor push against either side of the cavity and, most importantly, it causes unfavorable coumarin alignment. Accordingly, the R6-dimethylamine of derivatives **17**–**19** packs directly against the side chains of Phe103, Pro104, Trp119, Leu164, and Ile316 (Figures 4C,D, Supplementary Figure S3J).

### Refining the alignment via the R1–R3 substituents of the coumarin ring

Inserting a functional group such as methoxy to the R1/R2 position of the coumarin ring (Figure 2), capable of forming both hydrophobic and hydrophilic interactions, generally improves the MAO-B inhibition (Figure 4, Table 1).

The benefits of this sort of dual-purpose group are evident when comparing the activity of otherwise identical derivatives with and without the proximal group; i.e., **11**, that lacks only the R1-methoxy of **8** (Supplementary Figure S3F vs. Figure 5E), produces significantly lower inhibition (IC_50_ value of 798 vs. 231 nM; Table 1). On one hand, the methyl of the R1-methoxy group of **8** (Figure 5E) packs into a hydrophobic niche formed by the side chains of Tyr60, Gln206, Tyr326, Leu328, Phe343, and Met341 (green sector in Figures 3A,B). On the other hand, the methoxy's oxygen increases the 3-phenyl ring's hydrophilicity and softens the clash of the coumarin ring with the solvent shielding the cofactor (Figure 5E).

Switching the R1-methoxy of **1** into the R2 position in **2** (Figure 2) makes the alignment of the coumarin ring more challenging, because the R2-methoxy is unable to occupy the same hydrophobic niche (green sector in Figures 3A,B) as the R1-methoxy (Figure 4A vs. Supplementary Figure S3A). Although the R1/R2 methoxy switch, by all means, does not prevent binding, it leads to ~80 nM reduction in the IC_50_ value (Table 1). Paradoxically, the opposite and considerably larger difference in inhibition is produced by the R1/R2 switch, when comparing the activity of derivatives **20** and **22** (Figure 2; Table 1). Accordingly, **20** with the R2-methoxy of (IC_50_ value of 391 nM; Table 1) provides twice as strong inhibition as **22** with the R1-methoxy (IC_50_ value of 831 nM; Table 1). The vast difference is caused by the coordinated R6/R7 interactions of the 3-phenyl ring, which pushes the coumarin ring closer to the Tyr326 side chain—a critical shift that is stunted by the R1-methoxy of **22** (Figure 5E vs. Figure 5F).

Replacing the R2-acetoxy of **3** (Figure 2) with the R1-methoxy in **4** (Figure 2) weakens the inhibition ~180 nM (Table 1). The coumarin ring of **4** is pushed closer to the cofactor due to the addition of the R6-trifluoromethoxy into the 3-phenyl ring (Figure 5B vs. Supplementary Figure S3B) and, in this new pose, the methyl of the R2-acetoxy is able to occupy the small hydrophobic niche (green sector in Figures 3A,B), meanwhile, exposing the acetoxy's oxygen atoms to the solvent (Figure 3B). However, substituting the R1-methoxy of **18** with the R2-acetoxy in **19** (Figure 2) does not improve the inhibition; instead, the IC_50_ value is reduced by ~250 nM (Table 1). This happens, because the R6-dimethylamine of **19** (Supplementary Figure S3J) is not forcing the scaffold to align close to the cofactor the same way as the R6-trifluoromethoxy does (Figure 5B vs. Figures 6C,D). In contrast, replacing the R1-methoxy of **18** with the R2-hydroxyl in **17** improves the inhibition (IC_50_ improvement of 234 nM; Table 1) by promoting water solubility near the cofactor (Figure 6C vs. Figure 6D).

The R6 and R7 interactions of **7** (Figure 2) are expected to remind closely those of **6** (Supplementary Figure S3D vs. Figure 5C), but its coumarin ring's R1- and R3-chlorine groups weaken the inhibition ~700 nM (Table 1). The R2-methoxy of **6** is able to play into the hydrophobic/hydrophilic dual nature of the cavity end facing the cofactor (Figure 5C) without occupying the small hydrophobic niche (green sector in Figures 3A,B). In this respect, the R1-chlorine is too bulky to occupy this specific niche although a methoxy group at the same position should be able to occupy the available space (e.g., **1** in Figure 5A).

### Selectivity of the 3-phenylcoumarin derivatives

Determining the specificity and subtype selectivity of the 3-phenylcoumarin derivatives for MAO-B is needed to evaluate their true pharmacological potential. Unintended off-target effects with other proteins can render even the most promising drug candidates useless, ambiguous or even toxic. Here, the focus is put on MAO-A which has shared activity with MAO-B in deamination of dopamine and dietary amines tyramine and tryptamine. In addition, the effects of the derivatives are tested with a specific subset of enzymes, including HSD1, aromatase, CYP1A2, and ER, whose function is linked to different stages of estradiol action and metabolism. These particular enzymes were looked at with the derivatives, because they are known to have structurally similar ligands or even coumarin-based inhibitors based on prior studies and our upcoming study (Mattsson et al., 2014; Niinivehmas et al., 2016; Niinivehmas et al., unpublished results).

*Monoamine oxidase A (MAO-A)* is more prevalent than the subtype B in the gastrointestinal tract and, accordingly, the MAO-A inhibition can cause accumulation of tyramine from dietary sources. Because tyramine can displace neurotransmitters leading to potentially fatal hypertensive crisis, it is highly desirable to design MAO-B-specific inhibitors lacking MAO-A activity. The vast majority of the novel derivatives do not produce MAO-A inhibition at 100 μM despite the fact that it is ten times the concentration used in this study to determine MAO-B inhibition percentage (Table 1, Supplementary Table S1). Furthermore, only in those few cases where inhibition was detected, especially with the most potent MAO-B derivatives, it remains at moderate or close to non-existent level (Table 1). The strongest MAO-A inhibition was elicited by derivatives **42** and **43** (48.86 and 56.76%), but derivatives **27** and **45** (43.83 and 43.36%) are close runner-ups and next analogs down the list are already much weaker (Supplementary Figure S4, Supplementary Table S1). Notably, **1**, which is the most potent MAO-B inhibitor of the derivative set with the IC_50_ value of 56 nM, does not produce MAO-A inhibition at 100 μM (Table 1). The molecular basis for the lack of MAO-A activity is evident, when comparing the shape and size of the active sites of the two enzyme subtypes in the context of 3-phenylcoumarin binding (Figure 3A vs. Figure 3B).

*17-*β*-hydroxysteroid dehydrogenase 1 (HSD1)*, which functions as the catalyst of the final reducing step in the estradiol biosynthesis, is often overexpressed in breast cancer and endometriotic tissue (Vihko et al., 2004; Dassen et al., 2007; Hanamura et al., 2014). Thus, specific inhibition of HSD1 has potential to reduce effective estradiol levels in the treatments. Although the synthesized 3-phenylcoumarin set contains several molecules that exhibit activity toward HSD1, the inhibition was generally very weak and the active compounds are not among the most potent MAO-B inhibitors. Of the 24 most potent MAO-B inhibitors, the strongest HSD1 inhibition could be recorded for **20** and **22** (46 and 54%; Figure 2, Table 1); however, considerably higher activity (48.20–83.90%) was seen with derivatives **30**, **31**, **33**, **38**, and **48** (Supplementary Figure S4, Supplementary Table S1). Modest HSD1 inhibition (12–33%) was also elicited by **6**, **15**, **16**, **23**, **24** (Figure 2, Table 1) and **51** (Supplementary Figure S4, Supplementary Table S1). Importantly, derivative **1**, which is the most potent MAO-B inhibitor of the derivative set, does not inhibit HSD1.

*Aromatase (CYP19A1)* inhibition, which is important for blocking local estradiol synthesis for example in breast cancer treatment (Pasqualini et al., 1996), was not detected with the derivatives (Table 1, Supplementary Table S1). Although 3-phenylcoumarin should be able to sterically mimic the steroidal positioning at the active site (not shown), it would have to house a clear-cut H-bond acceptor at the R5/R7-position in the 3-phenyl to facilitate aromatase binding. This is, because X-ray crystallography shows that the Asp309 side chain is in neutral state at pH 7.4 and donating a proton to the carbonyl group of inhibitor androstenedione (PDB: 3EQM) (Ghosh et al., 2009). Inserting a hydroxyl group to the R5/R7 position could put an H-bond acceptor to this same location with the 3-phenylcoumarins (see **31**, **38**, **40**, **42**, **43**; Supplementary Figure S4, Supplementary Table S1). However, because the hydroxyl always has a dual role as an H-bond donor as well, any aromatase binding by the derivatives remains theoretical as it is prevented by a proton donor clash. The issue is described more thoroughly in our upcoming study (Niinivehmas et al., unpublished results).

*Estrogen receptor (ER)* agonists/antagonists or selective modulators are developed for infertility, contraception, hormone replacement, and ER positive breast cancer therapies. If the MAO-B inhibitors would function also as ER agonists, they could promote tumorigenesis in the breast tissue as a side effect. Unintended ER inhibition could also disturb natural estrogen levels or interrupt ER-targeted therapies. The measurements indicate that the 3-phenylcoumarin derivatives either are a hit or miss when considering ER inhibition. Although the ER activity could not be measured for all of the analogs due to running out of the synthesis products, the acquired results overwhelmingly support our prior findings stating that the R2-hydroxyl or the R6-hydroxyl/halogen is needed to prompt ER activity (Niinivehmas et al., 2016). This ER-specific effect is prominent with **12**, **20**, **22**, **27**, **28**, **29**, **30**, **39**, **40**, **41**, **44**, and **48** (Table 1, Supplementary Table S1, Figure 2, Supplementary Figure S4) and, moreover, ER activity is predicted for **17** and likely for **32** and **47** based on the well-established trend.

*Cytochrome P450 1A2 (CYP1A2)* catalyzes the oxidation of xenobiotics, especially polyaromatic hydrocarbons and steroid hormone-sized compounds such as 3-phenylcoumarins, into more soluble form for excretion (Zhou et al., 2010). Accordingly, it was prudent to get a rough estimate of the CYP1A2 inhibition levels for the novel 3-phenylcoumarin derivatives as well. In general, all of the derivatives inhibited CYP1A2 at some level (Table 1, Supplementary Table S1); however, typically the most potent CYP1A2 inhibitors such as **21**–**24** were less potent MAO-B inhibitors (Table 1). Similar to MAO-A, HSD1, and aromatase, the most potent MAO-B derivative **1** displayed only low CYP1A2 activity (IC_50_ value of 124 μM; Table 1).

### Overall assessment on the druglikeness

As a whole, the selectivity analysis indicates that the cross-reactivity of 3-phenylcoumarins can be managed or even avoided via specific functional group substitutions without taking away the MAO-B activity. Coumarins in general do not belong to the PAINS (pan assay interference compounds) category as it is a privileged scaffold structure. Only derivative **50**, which is not a potent MAO-B inhibitor (Supplementary Table S1, Supplementary Figure S4), was recognized as a potential PAINS ligand by PAINS3 filter (or A filter) in CANVAS module in MAESTRO (Baell and Holloway, 2010). In the ChEMBL database, ~14,200 coumarin derivatives are included (observed online in 8.2.2018), which indicates that the scaffold can be tailored to target multitude of proteins. Despite this, the literature does not raise widespread concerns that the coumarin-based compounds in particular would cause harmful cross-reactivity or selectivity issues. The 24 active derivatives presented in this study (Table 1, Figure 2) have lower potency than some of the prior 3-phenylcoumarin compounds (Supplementary Figure S6, Supplementary Table S2) (Matos et al., 2009b, 2011a,b; Santana et al., 2010; Viña et al., 2012a); however, one has to be aware of fact that these results originate from different laboratories and activity assays and are, therefore, not fully comparable. To a degree this is the case even for the positive control pargyline (Fisar et al., 2010). Importantly, the new compounds follow closely the Lipinski rule of five regarding the logP value (logP < 5) and remain in the logP range of 2–4. Moreover, the ligand-lipophilicity efficiency (LiPE) values of the new analogs suggest reasonable druglikeness (Freeman-Cook et al., 2013). What is more, derivative **1** clearly has the most promising selectivity profile of the derivatives for future consideration, because it is not only the most potent MAO-B inhibitor of the set but it is also selective against the other tested enzymes.

## Conclusion

A broad set of 3-phenylcoumarin derivatives was designed using virtual combinatorial chemistry or rationally *de novo*, synthesized and tested for MAO-B inhibition potency using spectrophotometry (Supplementary Table S1). The results further validate prior studies suggesting that the 3-phenylcoumarin is a suitable scaffold for building potent small-molecule MAO-B inhibitors by functionalizing its ring systems. A moderate MAO-B inhibition could be achieved by inserting a wide variety of functional groups into the coumarin (R1–R3; Figure 4) or 3-phenyl (R4–R7; Figure 4) rings (Supplementary Table S1). Twenty-four of the derivatives (Figures 2, 3D) were found to elicit >70% inhibition (Table 1, Supplementary Figures S1, S2). These promising derivatives inhibit the MAO-B at a ~100 nM to ~1 μM range (Table 1), while the most potent derivative **1** produces ~56 nM MAO-B inhibition. A molecular docking-based (Figures 5, 6, Supplementary Figure S3) SAR analysis (Figure 4) describe the determinants of the MAO-B binding and inhibition at the atomistic level. Firstly, without any kind of the 3-phenyl substituents, no inhibition was detected. Although both hydrogen and halogen bonding can assist the 3-phenyl alignment and facilitate inhibition (Figures 6E,F, Table 1), the ability of the functionalized ring to fill the hydrophobic end of the binding cavity (red sector in Figures 3A,B) is the most important property for ensuring strong MAO-B inhibition (e.g., R6-trifluoromethyl of **1**; Figure 5A). Secondly, the SAR analysis reveals that a spot-on placement and composition of the coumarin ring's substituents can further enhance the MAO-B inhibition (Figure 2, Table 1), however, these effects are ultimately dependent on the scaffold alignment, which, in turn, depends on the 3-phenyl ring substituents (Figure 4). The cross-reactivity analysis focusing on MAO-A and a subset of estradiol metabolism-linked HSD1, aromatase, CYP1A2 and ER highlighted the potential of the 3-phenylcourmains, especially the most potent MAO-B derivative **1**, for producing selective MAO-B inhibition. Finally, the most potent 3-phenylcoumarin analogs presented in this study are estimated to operate at close to optimal ligand-lipophilicity efficiency—a feature highlighting their overall druglikeness.

## Author contributions

SR: was responsible for the experimental testing regarding MAO-A and MAO-B; EMu: performed the MAO-A experimental analysis; SR: did the docking into MAO-B and prepared most of the figures; PAP: was responsible for the final SAR analysis; SK, ES, and JH: performed the organic synthesis; MA: did the PAINS screening; PK: performed the HSD1 measurements; NN, RJ, and HR: did the experimental analysis regarding CYP1A2; PH and MaP: executed the experimental analysis regarding aromatase; MiP: did preliminary screening for designing MAO-B ligands; SN, EMa, SK, and OTP: designed the molecules for the selected targets; SN, EMa, and OTP: designed the study. All the coauthors were involved in the manuscript preparation and approved the final version.

### Conflict of interest statement

The authors declare that the research was conducted in the absence of any commercial or financial relationships that could be construed as a potential conflict of interest.

## Abbreviations

MAO-A: monoamine oxidase A
MAO-B: monoamine oxidase B
HSD1 or 17-β-HSD1: 17-β-hydroxysteroid dehydrogenase 1
ER: estrogen receptor
CYP1A2: cytochrome P450 1A2
CYP19A1: aromatase
SAR: structure-activity relationship.
