# Supplementary material for: Structure-Activity Relationship Analysis of 3-Phenylcoumarin-Based Monoamine Oxidase B Inhibitors
Source: Front Chem. 2018 Mar 2;6:41. doi: 10.3389/fchem.2018.00041 (PMC5840146; doi:10.3389/fchem.2018.00041)
Supplement: Supplementary file 1 [file DataSheet1.docx]

Supplementary Material

Structure-Activity Relationship Analysis of 3-Phenylcoumarin-Based Monoamine Oxidase B Inhibitors

Sanna Rauhamäki, Pekka A. Postila, Sanna Niinivehmas, Sami Kortet, Emmi Schildt, Mira Pasanen, Elangovan Manivannan, Mira Ahinko, Pasi Koskimies, Niina Nyberg, Pasi Huuskonen, Elina Multamäki, Markku Pasanen, Risto O. Juvonen, Hannu Raunio, Juhani Huuskonen*, Olli T. Pentikäinen*

*** Correspondence:** Juhani Huuskonen: juhani.s-p.huuskonen@jyu.fi (synthesis),

Olli T. Pentikäinen: olli.pentikainen@utu.fi (modeling, *in vitro*)

# Supplementary Figures and Tables

## Supplementary Figures


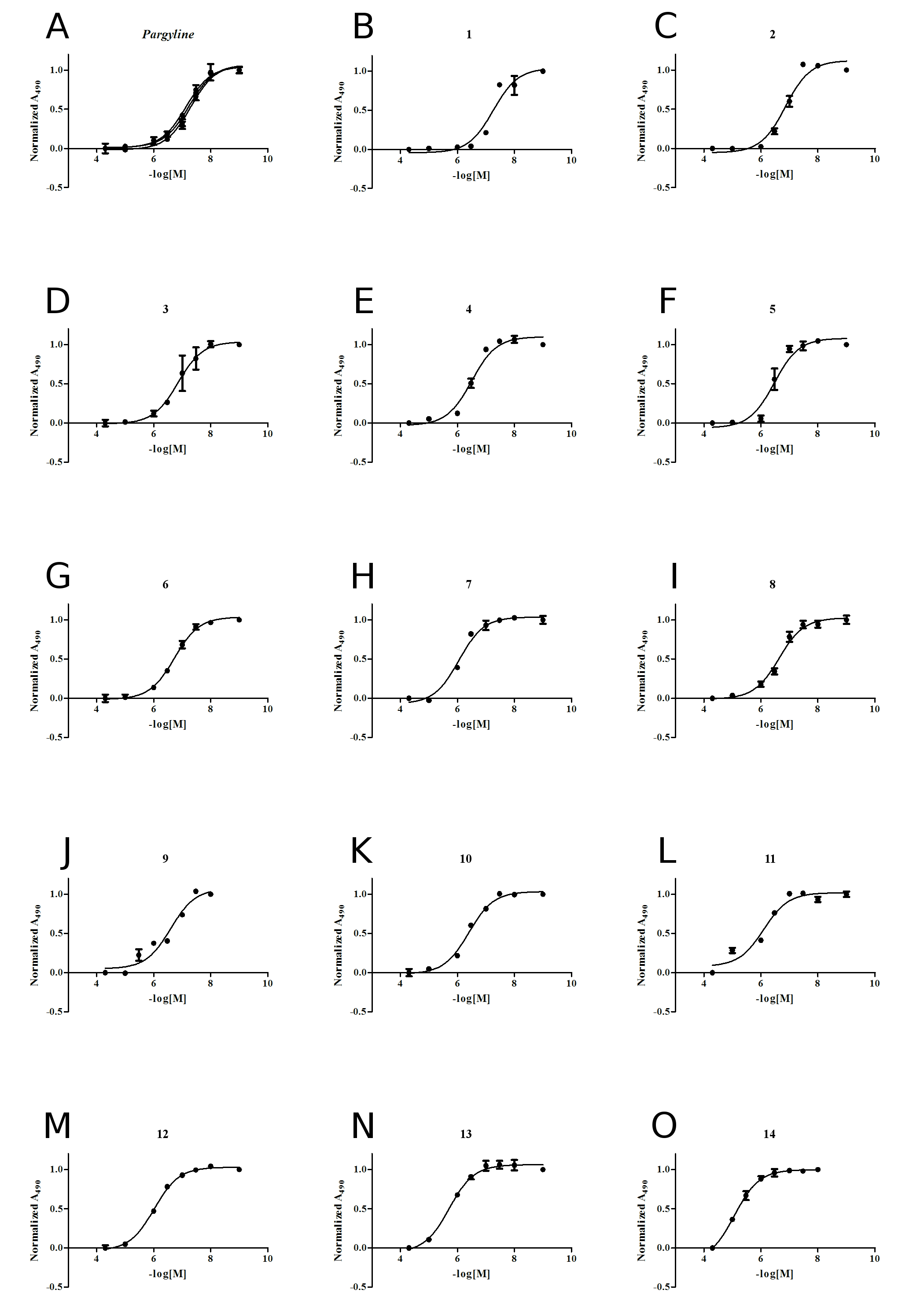


**Supplementary Figure 1.** MAO-B IC_50_ graphs for pargyline and 3-phenylcoumarin derivatives 1-14.


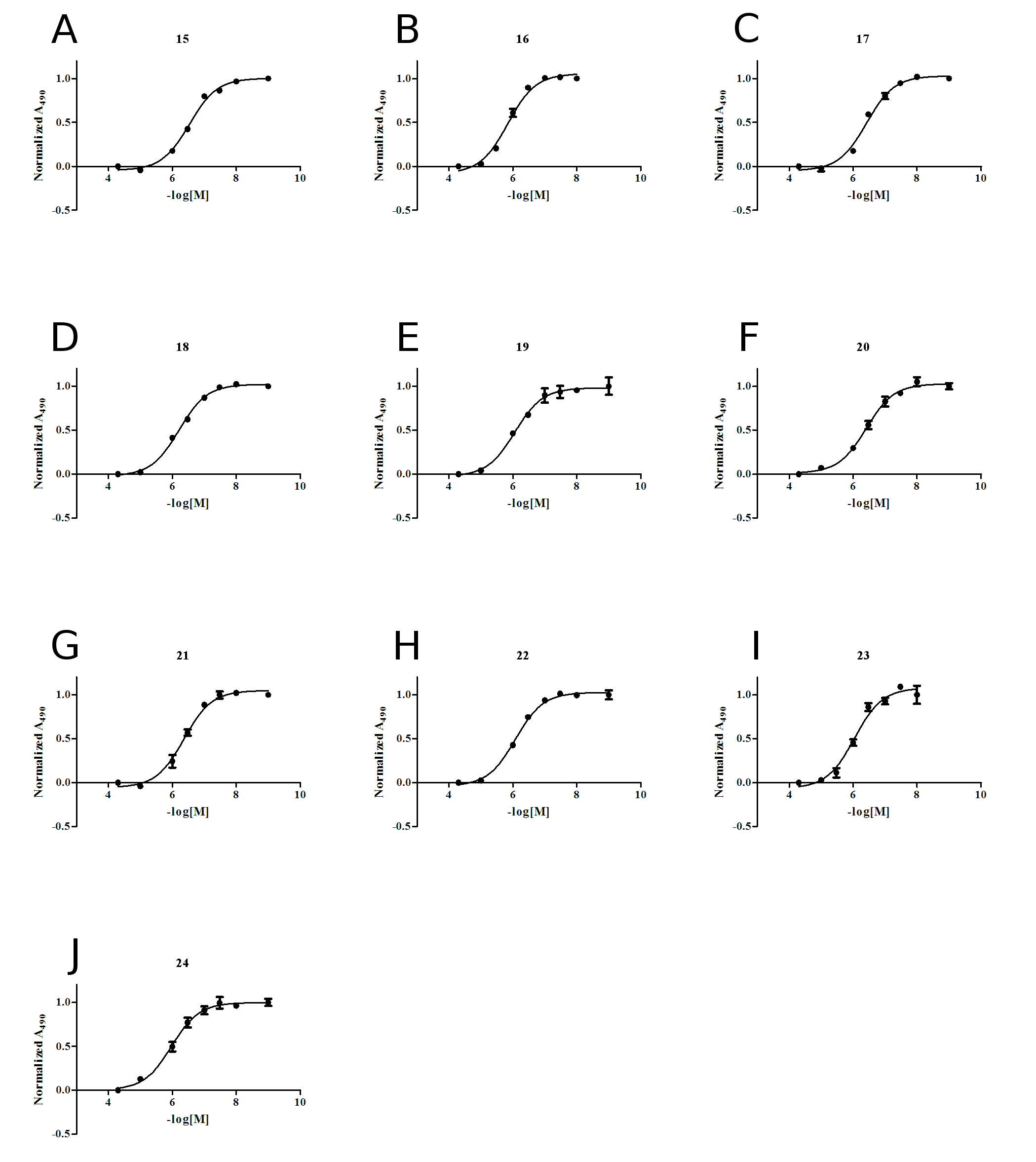


**Supplementary Figure 2.** MAO-B IC_50_ graphs for 3-phenylcoumarin derivatives 15-24.


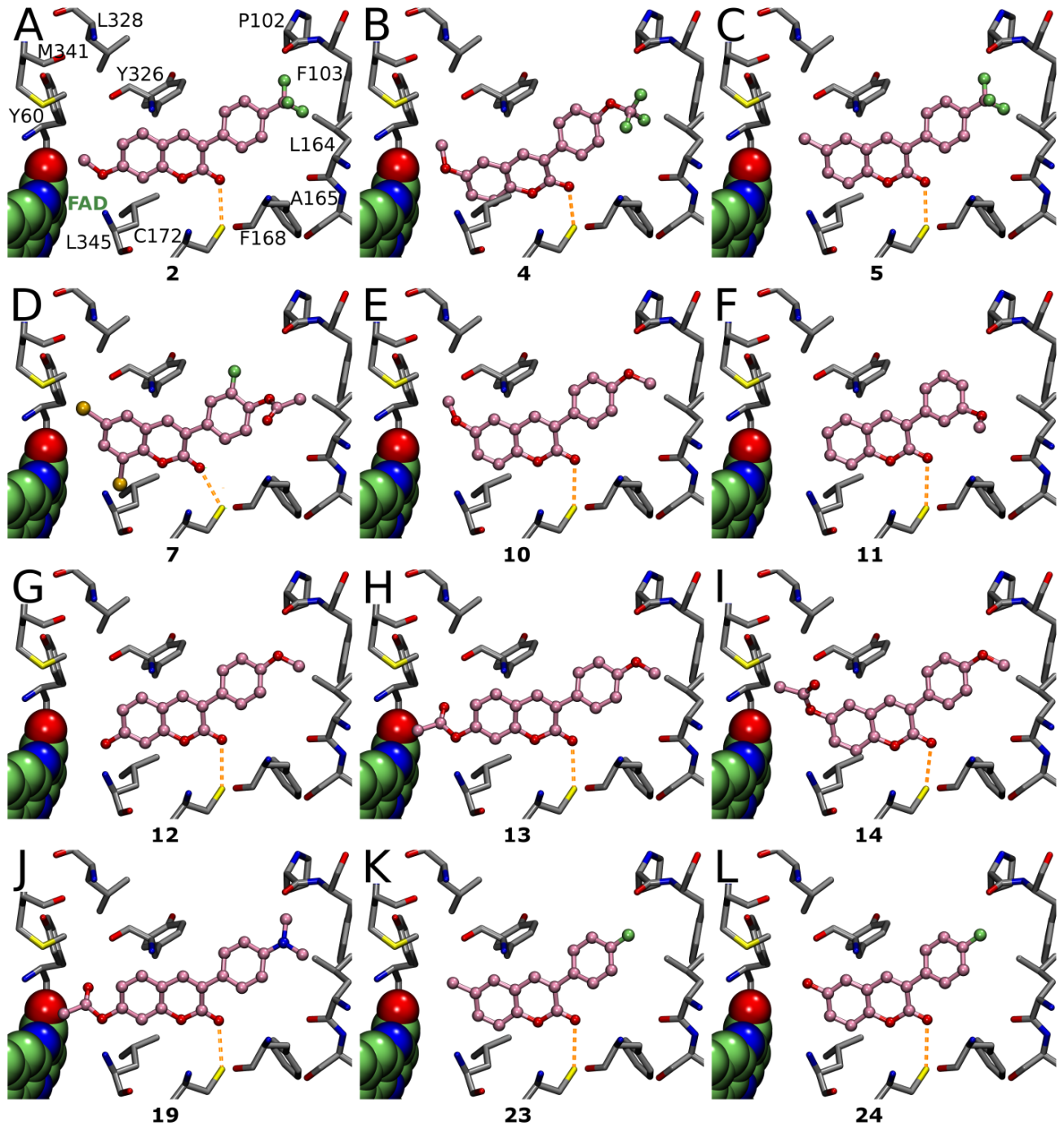


**Supplementary Figure 3.** The binding modes of selected 3-phenylcoumarin inhibitors. (A) Derivatives **2** (IC_50_ 138 nM), (B) **4** (IC_50_ 317 nM) and (C) **5** (IC_50_ 343 nM) have three fluorine atoms at R6 but the R2-methoxy of **2** ensures stronger MAO-B inhibition than the R1-methoxy of **4** or the R1-methyl of **5**. (D) Derivative **7** (IC_50_ 888 nM) has halogens on R1, R3 and R6 but it is hardly more active than (K) **23** (IC_50_ 902 nM). (E) Derivatives **10** (IC_50_ 400 nM), (G) **12** (IC_50_ 955 nM), (H) **13** (IC_50_ 1946 nM) and (I) **14** (IC_50_ 8476 nM) have methoxy on R6 and (F) **11** (IC_50_ 798 nM) has it on R7. Paired with methoxy on 3-phenyl, smaller substitutes like methoxy on R1 of **10** or hydroxyl on R2 of **12** are more suitable than acetoxy on R2 of **13** or on R1 of **14**. (J) Same applies on (J) **19** (IC_50_ 866 nM) which is less active than molecules **17** and **18** (Fig. 6) with the same R6. However, the smaller substitutes on (K) **23** (IC_50_ 902 nM) and (L) **24** (IC_50_ 1058 nM) do not compare to the suitable interactions of R1 methoxy of **21** (Fig. 5) or the combination of hydroxyl on R6 and halogen on R7 of **20** and **22** (Fig. 6). See Fig. 1, Fig. 2 and Table 1 for further details.

**
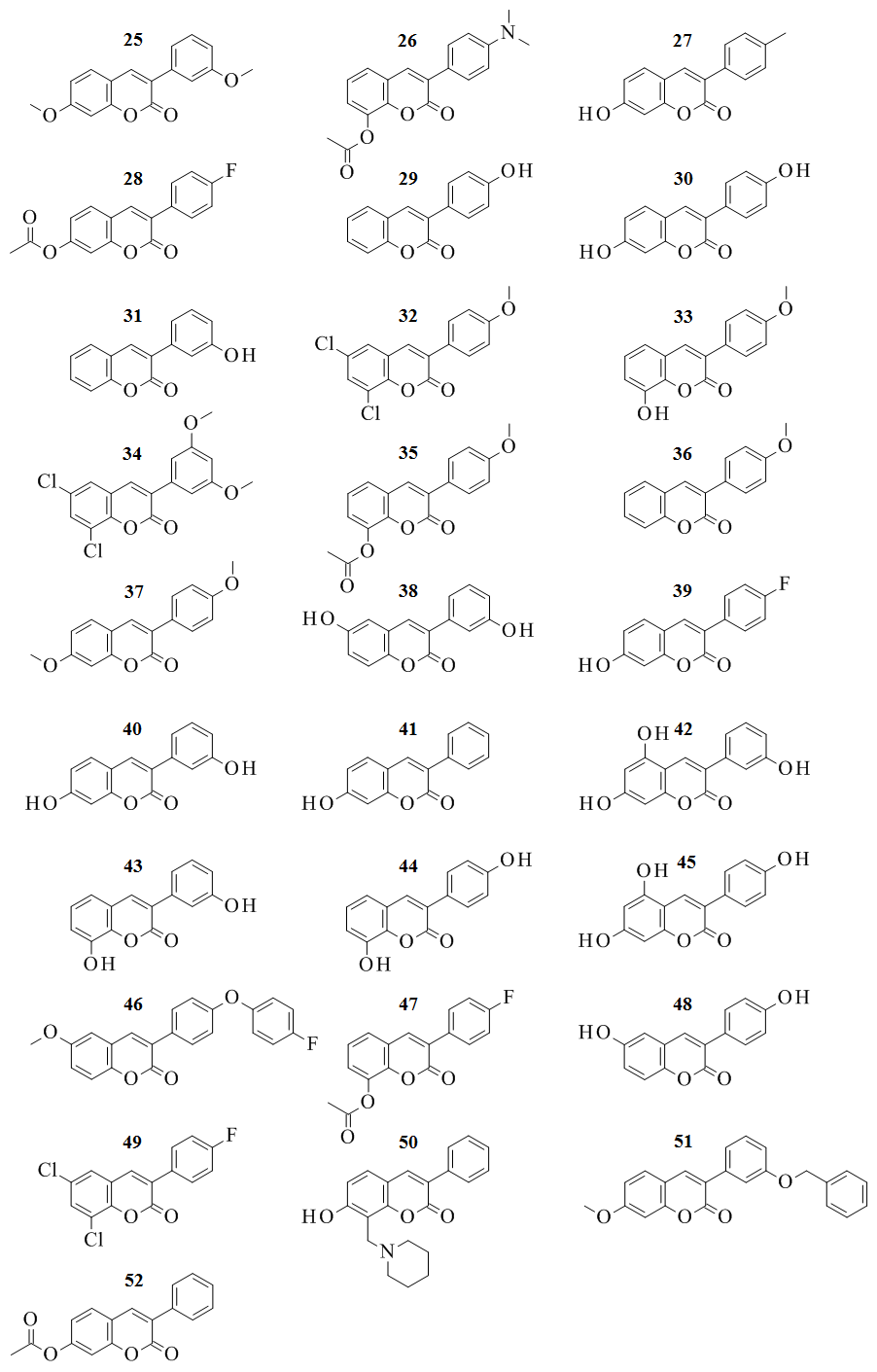
**

**Supplementary Figure 4.** The 3-phenylcoumarin derivatives 25-52. The compounds are put in descending order based on their MAO-B activity (Table S1).

**Supplementary Figure 5.** MAO-B and MAO-A activity in continuous spectrophotometric assay. The monoamine oxidase (MAO) activity was measured at A_490_ every 15 s, 300 times or until plateau was reached. MAO-A was able to induce significantly faster nearly double the absorbance with the selected dose used throughout the experiments in comparison to the MAO-B. The difference was taken into account in the duration of the experiment as the MAO activity was collected for data analysis at 1 h after the initiation of the assay reaction for MAO-A and at 2 h for MAO-B.

**
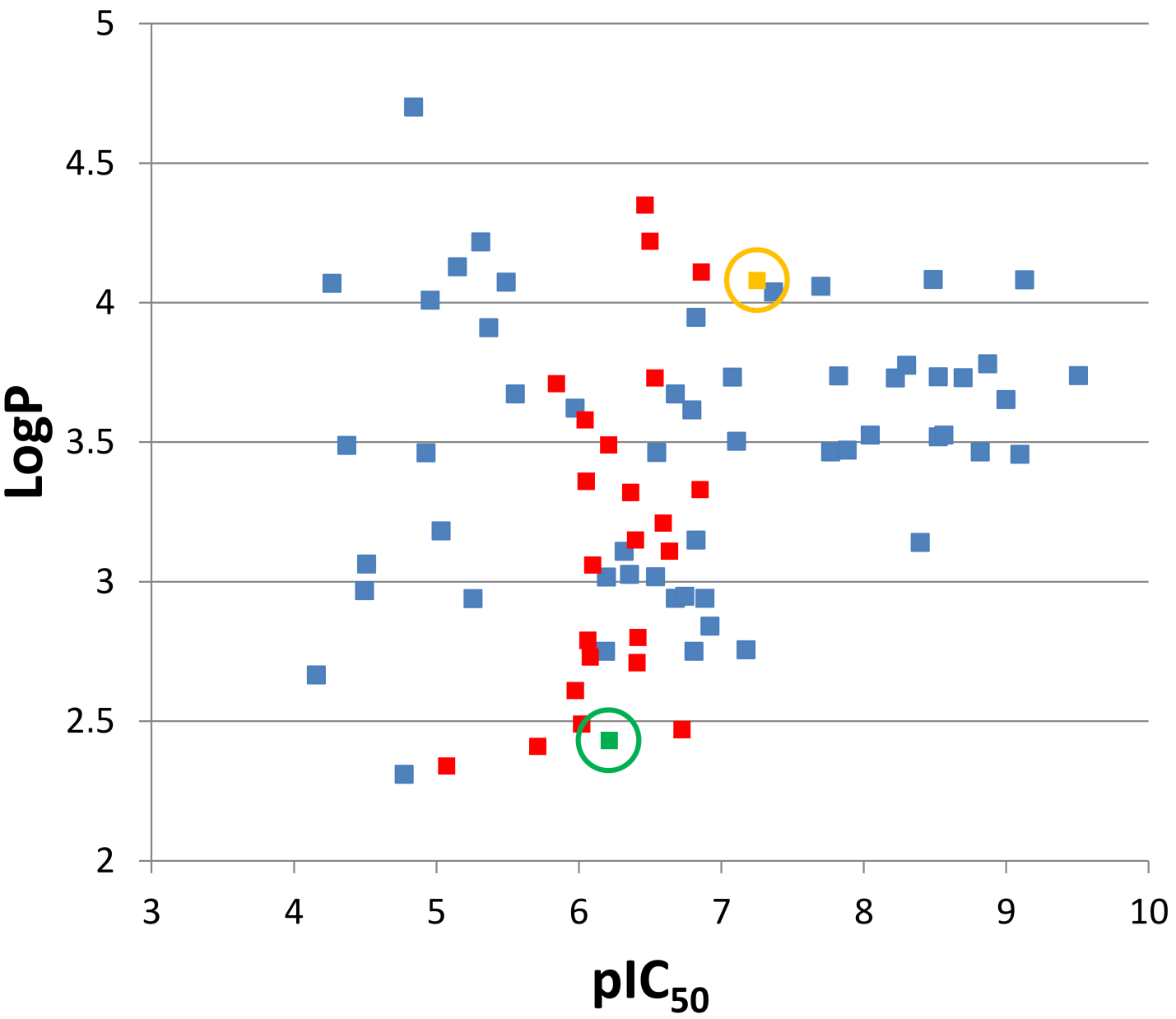
**

**Supplementary Figure 6.** The logP *versus* pIC_50_ value for 3-phenylcoumarin-based MAO-B inhibitors. The potency of the 24 novel analogs (red dots; Table 1; Fig. 2), prior 3-phenylcoumarins (blue dots) and the positive control pargyline (green dot) is plotted against the estimated lipophilicity (logP) of the compounds (Table S2). The most potent new derivative **1** (yellow dot) and the pargyline are highlighted with yellow and green circles.

## Supplementary Tables

**Supplementary Table 1.** Inhibition data on the 3-phenylcoumarin derivatives 25-52.

| **ID** | **QPlogPo/w** | **MAO-A inhibition %**  **(100 µM, 1h)** | **MAO-B inhibition %**  **(10 µM, 2h)** | **ER inhibition % (10 µM)** | **HSD1 inhibition %**  **(1 µM)** | **CYP1A2 inhibition IC_50_ µM** |
| --- | --- | --- | --- | --- | --- | --- |
| **25** | 3.16 | 0.00 | 67.49 | 0.00 | 3.50 | 35.00 |
| **26** | 2.56 | 0.00 | 66.79 | N/A | 0.00 | 7.00 |
| **27** | 2.70 | 43.83 | 61.91 | 70.96 | 12.50 | 180.00 |
| **28** | 2.64 | 0.00 | 59.20 | 57.12 | 0.30 | 114.00 |
| **29**^(*)^ | 2.40 | 0.00 | 53.65 | 74.22 | 5.40 | 26.00 |
| **30**^(*)^ | 1.67 | 0.00 | 52.68 | 58.53 | 48.20 | 22.00 |
| **31**^(*)^ | 2.37 | 4.14 | 49.60 | 0.00 | 56.00 | 7.00 |
| **32**^(*)^ | 4.10 | 0.00 | 47.77 | N/A | N/A | 800.00 |
| **33** | 2.51 | 3.66 | 47.64 | 0.00 | 76.70 | 86.00 |
| **34** | 4.15 | 0.00 | 46.01 | N/A | 0.30 | 30.00 |
| **35** | 2.40 | 4.08 | 42.37 | N/A | 0.00 | 26.00 |
| **36**^(*)^ | 3.09 | 0.87 | 41.12 | 0.00 | 3.80 | 26.00 |
| **37** | 3.15 | 0.00 | 39.78 | N/A | 0.00 | 480.00 |
| **38**^(*)^ | 1.65 | 13.11 | 27.16 | 18.53 | 49.10 | 24.00 |
| **39**^(*)^ | 2.62 | 12.56 | 26.44 | 100.63 | 0.00 | 240.00 |
| **40** | 1.65 | 0.00 | 25.74 | 18.58 | N/A | N/A |
| **41**^(*)^ | 2.38 | 9.52 | 23.82 | 101.40 | N/A | 8.00 |
| **42** | 1.01 | 48.86 | 23.30 | 0.44 | N/A | N/A |
| **43** | 1.70 | 56.76 | 22.93 | 0.00 | N/A | N/A |
| **44**^(*)^ | 1.70 | 22.39 | 22.52 | 96.18 | N/A | N/A |
| **45** | 1.01 | 43.38 | 21.38 | 0.13 | N/A | N/A |
| **46** | 4.76 | 8.11 | 19.73 | 0.00 | 0.00 | 240.00 |
| **47** | 2.46 | 22.33 | 15.81 | N/A | 0.00 | 17.00 |
| **48**^(*)^ | 1.66 | 0.00 | 15.50 | 98.41 | 83.90 | 33.00 |
| **49** | 4.29 | 0.00 | 10.23 | 0.66 | 1.30 | N/A |
| **50** | 3.21 | 0.00 | 8.16 | N/A | 1.50 | 65.00 |
| **51** | 4.75 | 0.00 | 6.66 | N/A | 31.60 | 3.00 |
| **52**^(*)^ | 2.43 | 0.00 | 0.00 | N/A | 0.70 | 80.00 |

N/A = not available. ^(*)^ Compounds **29** (Leitão et al., 2004)**, 30** (Yang et al., 2011)**, 31** (Leitão et al., 2004)**, 32** (Pu et al., 2014)**, 36** (Pu et al., 2014)**, 38** (Kabeya et al., 2008)**, 39** (CHEMBL1387945), **41** (Wang et al., 2011)**, 44** (Matos et al., 2013a)**, 48** (Kabeya et al., 2008) and **52** (CHEMBL1559283) had been synthesized independently prior to this study but not tested for MAO-B activity. The compounds are in descending order (**25**-**52**) based on their MAO-B activity.

**Supplementary Table 2.** MAO-B inhibition and lipophilicity data on new and prior 3-phenylcoumarins.

| **ID** | **MAO-B inhibition IC_50_ nM** | **QPlogPo/w^(1)^** | **pIC_50_** | **LiPE^(2)^** | **Reference** |
| --- | --- | --- | --- | --- | --- |
| **Pargyline** | 61 | 2.431 | 6.21 | 3.78 |  |
| **01** | 56 | 4.08 | 7.25 | 3.17 |  |
| **02** | 138 | 4.11 | 6.86 | 2.75 |  |
| **03** | 141 | 3.33 | 6.85 | 3.52 | (Dobelmann-Mara et al., 2017) |
| **04** | 317 | 4.22 | 6.50 | 2.28 |  |
| **05** | 343 | 4.35 | 6.46 | 2.11 |  |
| **06** | 189 | 2.47 | 6.72 | 4.25 |  |
| **07** | 888 | 3.36 | 6.05 | 2.69 |  |
| **08** | 231 | 3.11 | 6.64 | 3.53 |  |
| **09** | 255 | 3.21 | 6.59 | 3.38 | (Vilar et al., 2006) |
| **10** | 400 | 3.15 | 6.40 | 3.25 | (Ferino et al., 2013; Prendergast, 2001) |
| **11** | 798 | 3.06 | 6.10 | 3.04 | (Kirkiacharian et al., 1999) |
| **12** | 955 | 2.49 | 6.02 | 3.53 | (Prendergast, 2001) |
| **13** | 1946 | 2.41 | 5.71 | 3.30 | (Bhandri et al., 1949) |
| **14** | 8476 | 2.34 | 5.07 | 2.73 |  |
| **15** | 292 | 3.73 | 6.53 | 2.80 |  |
| **16** | 1433 | 3.71 | 5.84 | 2.13 |  |
| **17** | 384 | 2.8 | 6.42 | 3.62 | (Kirkiacharian et al., 2003) |
| **18** | 617 | 3.49 | 6.21 | 2.72 |  |
| **19** | 866 | 2.79 | 6.06 | 3.27 |  |
| **20** | 391 | 2.71 | 6.41 | 3.70 |  |
| **21** | 433 | 3.32 | 6.36 | 3.04 |  |
| **22** | 831 | 2.73 | 6.08 | 3.35 |  |
| **23** | 902 | 3.58 | 6.04 | 2.46 | (Chauhan et al., 2016) |
| **24** | 1058 | 2.61 | 5.98 | 3.37 |  |
| **M227^(3)^** | 440 | 3.03 | 6.36 | 3.33 | (Santana et al., 2010) |
| **M228^(3)^** | 210 | 2.94 | 6.68 | 3.74 | (Santana et al., 2010) |
| **M229^(3)^** | 130 | 2.94 | 6.89 | 3.95 | (Santana et al., 2010) |
| **M230^(3)^** | N/A | 3.72 | N/A | N/A | (Santana et al., 2010) |
| **M231^(3)^** | 1 | 3.65 | 9.00 | 5.35 | (Santana et al., 2010) |
| **M232^(3)^** | 4 | 3.14 | 8.40 | 5.26 | (Santana et al., 2010) |
| **M233^(3)^** | 480 | 3.11 | 6.32 | 3.21 | (Santana et al., 2010) |
| **M234^(3)^** | 290 | 3.02 | 6.54 | 3.52 | (Santana et al., 2010) |
| **M235^(3)^** | 640 | 3.02 | 6.19 | 3.18 | (Santana et al., 2010) |
| **M236^(3)^** | N/A | 3.80 | N/A | N/A | (Santana et al., 2010) |
| **M237^(3)^** | 2 | 3.73 | 8.70 | 4.97 | (Santana et al., 2010) |
| **M238^(3)^** | 6 | 3.73 | 8.22 | 4.49 | (Santana et al., 2010) |
| **M243^(3)^** | 43 | 4.04 | 7.37 | 3.33 | (Santana et al., 2010) |
| **M271^(3)^** | 78 | 3.50 | 7.11 | 3.60 | (Viña et al., 2012a) |
| **M272^(3)^** | 3 | 3.52 | 8.52 | 5.00 | (Viña et al., 2012a) |
| **M273^(3)^** | 5 | 3.78 | 8.30 | 4.53 | (Viña et al., 2012a) |
| **M274^(3)^** | 20 | 4.06 | 7.70 | 3.64 | (Viña et al., 2012a) |
| **M275^(3)^** | 150 | 3.15 | 6.82 | 3.67 | (Viña et al., 2012a) |
| **M276^(3)^** | 3 | 3.73 | 8.52 | 4.79 | (Viña et al., 2012a) |
| **M277^(3)^** | 1060 | 3.62 | 5.97 | 2.35 | (Viña et al., 2012a) |
| **M278^(3)^** | 210 | 3.67 | 6.68 | 3.01 | (Viña et al., 2012a) |
| **M279^(3)^** | 150 | 3.95 | 6.82 | 2.88 | (Viña et al., 2012a) |
| **CHEMBL1221929** | 4300 | 3.91 | 5.37 | 1.46 | (Matos et al., 2010) |
| **CHEMBL1221930** | 11050 | 4.01 | 4.96 | 0.95 | (Matos et al., 2010) |
| **CHEMBL1221980** | 3230 | 4.07 | 5.49 | 1.42 | (Matos et al., 2010) |
| **CHEMBL1221981** | 7120 | 4.13 | 5.15 | 1.02 | (Matos et al., 2010) |
| **CHEMBL1221982** | 4890 | 4.22 | 5.31 | 1.09 | (Matos et al., 2010) |
| **CHEMBL1777814** | 83.48 | 3.73 | 7.08 | 3.35 | (Matos et al., 2011b) |
| **CHEMBL1777845** | 1.35 | 3.78 | 8.87 | 5.09 | (Matos et al., 2011b) |
| **CHEMBL1777847** | 30910 | 3.06 | 4.51 | 1.45 | (Matos et al., 2011b) |
| **CHEMBL1777849** | 16870 | 2.31 | 4.77 | 2.46 | (Matos et al., 2011b) |
| **CHEMBL1783714** | 283.75 | 3.46 | 6.55 | 3.08 | (Matos et al., 2009, 2011a; Viña et al., 2012b) |
| **CHEMBL1783715** | 8.98 | 3.53 | 8.05 | 4.52 | (Matos et al., 2009, 2011a) |
| **CHEMBL1783716** | 160.64 | 3.62 | 6.79 | 3.18 | (Matos et al., 2009, 2011a) |
| **CHEMBL1835223** | 17.05 | 3.47 | 7.77 | 4.30 | (Matos et al., 2011a) |
| **CHEMBL1835224** | 1.52 | 3.47 | 8.82 | 5.35 | (Matos et al., 2011a) |
| **CHEMBL1835225** | 67.1 | 2.76 | 7.17 | 4.42 | (Matos et al., 2011a) |
| **CHEMBL1835226** | 5520 | 2.94 | 5.26 | 2.32 | (Matos et al., 2011a) |
| **CHEMBL1835227** | 14470 | 4.70 | 4.84 | 0.14 | (Matos et al., 2011a) |
| **CHEMBL1835228** | 0.31 | 3.74 | 9.51 | 5.77 | (Matos et al., 2011a; Viña et al., 2012b) |
| **CHEMBL1835229** | 15.01 | 3.74 | 7.82 | 4.09 | (Matos et al., 2011a) |
| **CHEMBL1835230** | 2.73 | 3.53 | 8.56 | 5.04 | (Matos et al., 2011a) |
| **CHEMBL1835231** | 0.74 | 4.08 | 9.13 | 5.05 | (Matos et al., 2011a) |
| **CHEMBL1835232** | 3.25 | 4.08 | 8.49 | 4.41 | (Matos et al., 2011a) |
| **CHEMBL1835233** | 54030 | 4.07 | 4.27 | 0.20 | (Matos et al., 2011a) |
| **CHEMBL1835234** | N/A | 4.11 | N/A | N/A | (Matos et al., 2011a) |
| **CHEMBL1835326** | 650.03 | 2.75 | 6.19 | 3.44 | (Matos et al., 2011a) |
| **CHEMBL1835327** | 120.02 | 2.84 | 6.92 | 4.08 | (Matos et al., 2011a) |
| **CHEMBL1835328** | 180.04 | 2.95 | 6.74 | 3.80 | (Matos et al., 2011a) |
| **CHEMBL1917495** | 69590 | 2.67 | 4.16 | 1.49 | (Serra et al., 2012) |
| **CHEMBL1934673** | 32040 | 2.97 | 4.49 | 1.53 | (Serra et al., 2012) |
| **CHEMBL1934674** | N/A | 3.15 | N/A | N/A | (Serra et al., 2012) |
| **CHEMBL1934675** | 9260 | 3.18 | 5.03 | 1.85 | (Serra et al., 2012) |
| **CHEMBL1934676** | 42680 | 3.49 | 4.37 | 0.88 | (Serra et al., 2012) |
| **CHEMBL1934677** | 2790 | 3.67 | 5.55 | 1.88 | (Serra et al., 2012) |
| **CHEMBL510349** | 11810 | 3.46 | 4.93 | 1.47 | (Viña et al., 2012b) |
| **CHEMBL570703** | N/A | 3.54 | N/A | N/A | (Matos et al., 2011a) |
| **CHEMBL570731** | 0.8 | 3.46 | 9.10 | 5.64 | (Matos et al., 2011a) |
| **CHEMBL572233** | 13.05 | 3.47 | 7.88 | 4.41 | (Matos et al., 2009, 2011a) |
| **CHEMBL577099** | 155.59 | 2.75 | 6.81 | 4.06 | (Matos et al., 2011a) |
| **CHEMBL64744** | N/A | 2.57 | N/A | N/A | (Serra et al., 2012) |

N/A = not available. ^(1)^ QPlogPo/w calculated using Schrödinger Release 2017-1: QikProp (Schrödinger, LLC, New York, NY, 2017). ^(2)^ LiPE = pIC_50_ - QPlogPo/w. ^(3)^ Numbered as in Matos *et al.* (2013) (Matos et al., 2013b) and designated with letter M. Naming of the rest of the compounds follows the ChEMBL (<https://www.ebi.ac.uk/chembl/>).

# References

Bhandri, P. R., Bose, J. L., and Siddiqui, S. (1949). 3-​Phenylcoumarin series. I. Synthesis of some new members of the series. *J. Sci. Ind. Res.* 8B, 189–192.

Chauhan, P., Ravi, M., Singh, S., Prajapati, P., and Yadav, P. P. (2016). Regioselective [small alpha]-arylation of coumarins and 2-pyridones with phenylhydrazines under transition-metal-free conditions. *RSC Adv.* 6, 109–118. doi:10.1039/C5RA20954D.

Dobelmann-Mara, L., Riedmueller, S., and Schraub, M. (2017). Compounds for optically active devices. *PCT Int. Appl.*, A1 20170302.

Ferino, G., Cadoni, E., Matos, M. J., Quezada, E., Uriarte, E., Santana, L., et al. (2013). MAO Inhibitory Activity of 2-Arylbenzofurans versus 3-Arylcoumarins: Synthesis, in vitro Study, and Docking Calculations. *ChemMedChem* 8, 956–966. doi:10.1002/cmdc.201300048.

Kabeya, L. M., da Silva, C. H. T. P., Kanashiro, A., Campos, J. M., Azzolini, A. E. C. S., Polizello, A. C. M., et al. (2008). Inhibition of immune complex-mediated neutrophil oxidative metabolism: A pharmacophore model for 3-phenylcoumarin derivatives using GRIND-based 3D-QSAR and 2D-QSAR procedures. *Eur. J. Med. Chem.* 43, 996–1007. doi:10.1016/j.ejmech.2007.07.003.

Kirkiacharian, S., Chidiack, H., Philibert, D., Van De Velde, P., and Bouchoux, F. (1999). Binding affinity to steroid hormone receptors and antiproliferative action on MCF-​7 cells of coumarinic derivatives and isoflavonoids. *Ann. Pharm. Fr.* 57, 332–339.

Kirkiacharian, S., Lormier, A. T., Resche-Rigon, M., Bouchoux, F., and Cerede, E. (2003). Synthesis and binding affinity of 3-aryl-7-hydroxycoumarins to human α and β estrogen receptors. *Ann. Pharm. Fr.* 61, 51–56. doi:APF-01-2003-61-1-0003-4509-101019-ART2.

Leitão, A., Andricopulo, A. D., Oliva, G., Pupo, M. T., de Marchi, A. a, Vieira, P. C., et al. (2004). Structure-activity relationships of novel inhibitors of glyceraldehyde-3-phosphate dehydrogenase. *Bioorg. Med. Chem. Lett.* 14, 2199–2204. doi:10.1016/j.bmcl.2004.02.025.

Matos, M. J., Pérez-Cruz, F., Vazquez-Rodriguez, S., Uriarte, E., Santana, L., Borges, F., et al. (2013a). Remarkable antioxidant properties of a series of hydroxy-3-arylcoumarins. *Bioorganic Med. Chem.* 21, 3900–3906. doi:10.1016/j.bmc.2013.04.015.

Matos, M. J., Terán, C., Pérez-Castillo, Y., Uriarte, E., Santana, L., and Viña, D. (2011a). Synthesis and study of a series of 3-arylcoumarins as potent and selective monoamine oxidase B inhibitors. *J. Med. Chem.* 54, 7127–7137. doi:10.1021/jm200716y.

Matos, M. J., Vazquez-Rodriguez, S., Uriarte, E., Santana, L., and Viña, D. (2011b). MAO inhibitory activity modulation: 3-Phenylcoumarins versus 3-benzoylcoumarins. *Bioorganic Med. Chem. Lett.* 21, 4224–4227. doi:10.1016/j.bmcl.2011.05.074.

Matos, M. J., Viña, D., Janeiro, P., Borges, F., Santana, L., and Uriarte, E. (2010). New halogenated 3-phenylcoumarins as potent and selective MAO-B inhibitors. *Bioorganic Med. Chem. Lett.* 20, 5157–5160. doi:10.1016/j.bmcl.2010.07.013.

Matos, M. J., Viña, D., Quezada, E., Picciau, C., Delogu, G., Orallo, F., et al. (2009). A new series of 3-phenylcoumarins as potent and selective MAO-B inhibitors. *Bioorg. Med. Chem. Lett.* 19, 3268–3270. doi:10.1016/j.bmcl.2009.04.085.

Matos, M. J., Viña, D., Vazquez-Rodriguez, S., Uriarte, E., and Santana, L. (2013b). Focusing on New Monoamine Oxidase Inhibitors: Differently Substituted Coumarins As An Interesting Scaffold. *Curr. Top. Med. Chem.* 12, 2210–2239. doi:10.2174/1568026611212200008.

Prendergast, P. T. (2001). Use of flavones, coumarins and related compounds to treat infections. *PCT Int. Appl.*, A2 20010118.

Pu, W., Lin, Y., Zhang, J., Wang, F., Wang, C., and Zhang, G. (2014). 3-Arylcoumarins: Synthesis and potent anti-inflammatory activity. *Bioorg. Med. Chem. Lett.* 24, 5432–5434. doi:10.1016/j.bmcl.2014.10.033.

Santana, L., Orallo, F., Viña, D., Matos Joao Correia, P. C., Quezada, E., Yañes, J., et al. (2010). Use of derivates of 6-substituted 3-phenylcoumarins and preparation of new derivates. Available at: http://www.google.com.pg/patents/WO2010086484A1?cl=en.

Serra, S., Ferino, G., Matos, M. J., Vázquez-Rodríguez, S., Delogu, G., Viña, D., et al. (2012). Hydroxycoumarins as selective MAO-B inhibitors. *Bioorganic Med. Chem. Lett.* 22, 258–261. doi:10.1016/j.bmcl.2011.11.020.

Vilar, S., Quezada, E., Santana, L., Uriarte, E., Yánez, M., Fraiz, N., et al. (2006). Design, synthesis, and vasorelaxant and platelet antiaggregatory activities of coumarin–resveratrol hybrids. *Bioorg. Med. Chem. Lett.* 16, 257–261. doi:10.1016/j.bmcl.2005.10.013.

Viña, D., Matos, M. J., Ferino, G., Cadoni, E., Laguna, R., Borges, F., et al. (2012a). 8-Substituted 3-Arylcoumarins as Potent and Selective MAO-B Inhibitors: Synthesis, Pharmacological Evaluation, and Docking Studies. *ChemMedChem* 7, 464–470. doi:10.1002/cmdc.201100538.

Viña, D., Matos, M. J., Yáñez, M., Santana, L., and Uriarte, E. (2012b). 3-Substituted coumarins as dual inhibitors of AChE and MAO for the treatment of Alzheimer’s disease. *Med. Chem. Commun.* 3, 213–218. doi:10.1039/C1MD00221J.

Wang, C., Wu, C., Zhu, J., Miller, R. H., and Wang, Y. (2011). Design, Synthesis and Evaluation of Coumarin-based Molecular Probes for Imaging of Myelination. *J. Med. Chem.* 14, 2331–2340. doi:10.1016/j.jacc.2007.01.076.White.

Yang, J., Liu, G. Y., Dai, F., Cao, X. Y., Kang, Y. F., Hu, L. M., et al. (2011). Synthesis and biological evaluation of hydroxylated 3-phenylcoumarins as antioxidants and antiproliferative agents. *Bioorganic Med. Chem. Lett.* 21, 6420–6425. doi:10.1016/j.bmcl.2011.08.090.
